# Improved Method for Linear B-Cell Epitope Prediction Using Antigen’s Primary Sequence

**DOI:** 10.1371/journal.pone.0062216

**Published:** 2013-05-07

**Authors:** Harinder Singh, Hifzur Rahman Ansari, Gajendra P. S. Raghava

**Affiliations:** Bioinformatics Center, Institute of Microbial Technology, Chandigarh, India; Kyushu Institute of Technology, Japan

## Abstract

One of the major challenges in designing a peptide-based vaccine is the identification of antigenic regions in an antigen that can stimulate B-cell’s response, also called B-cell epitopes. In the past, several methods have been developed for the prediction of conformational and linear (or continuous) B-cell epitopes. However, the existing methods for predicting linear B-cell epitopes are far from perfection. In this study, an attempt has been made to develop an improved method for predicting linear B-cell epitopes. We have retrieved experimentally validated B-cell epitopes as well as non B-cell epitopes from Immune Epitope Database and derived two types of datasets called Lbtope_Variable and Lbtope_Fixed length datasets. The Lbtope_Variable dataset contains 14876 B-cell epitope and 23321 non-epitopes of variable length where as Lbtope_Fixed length dataset contains 12063 B-cell epitopes and 20589 non-epitopes of fixed length. We also evaluated the performance of models on above datasets after removing highly identical peptides from the datasets. In addition, we have derived third dataset Lbtope_Confirm having 1042 epitopes and 1795 non-epitopes where each epitope or non-epitope has been experimentally validated in at least two studies. A number of models have been developed to discriminate epitopes and non-epitopes using different machine-learning techniques like Support Vector Machine, and K-Nearest Neighbor. We achieved accuracy from ∼54% to 86% using diverse s features like binary profile, dipeptide composition, AAP (amino acid pair) profile. In this study, for the first time experimentally validated non B-cell epitopes have been used for developing method for predicting linear B-cell epitopes. In previous studies, random peptides have been used as non B-cell epitopes. In order to provide service to scientific community, a web server LBtope has been developed for predicting and designing B-cell epitopes (http://crdd.osdd.net/raghava/lbtope/).

## Introduction

Identification of smallest regions in an antigen also called an antigenic region that can activate immune system is one of the major challenges in designing of a subunit or peptide-based vaccine. These antigenic regions, which stimulate B-cell response, are known as B-cell epitopes. Prediction of B-cell epitope is difficult but important for designing a peptide-based vaccine [1]. B-cell epitopes can be divided in two categories (i) continuous and (ii) discontinuous. The continuous or linear epitopes are made up of consecutive amino acids whereas the discontinuous or conformational epitopes constitute the spatially folded amino acids, which lie far away in the primary sequence. Linear B-cell epitope has vast application in the area of antibody production, immunodiagnostics; epitope-based vaccine design, selective de-immunization of therapeutic proteins and in autoimmunity [2], [3], [4]. Experimental methods for identification of B-cell epitopes are costly and time consuming.

In order to overcome limitations of experimental techniques, in the past several algorithms have been developed to predict linear B-cell epitopes [5], [6]. Due to variability in epitope length from 3–85 amino acids, the prediction of B-cell epitopes is much more complex than the prediction of T-cell epitopes. Recently, Kringelum *et al*, have analyzed conformational B-cell epitopes from antigen-antibody complexes and reported average length of conformation epitope as 15 residues [7]. Though the average length is 15, it does not mean that B-cell epitope core has 15 residues. To the best of author’s knowledge it is not known that what, is the minimum length of a conformational or continuous B-cell epitope. All methods developed so far have two major limitations, (i) they are based on a very limited number of epitopes (∼1000 epitopes and non-epitopes) and second (ii) these methods use random peptides as non B-cell epitope [8], [9], [10], [11], [12] (Table S1).

In this study, for the first time, we have exploited the availability of several thousands of experimentally verified epitopes and non-epitopes. We have derived five datasets from Immune Epitope Database (IEDB) called Lbtope_Fixed, Lbtope_Fixed_non_redundant, Lbtope_Variable, Lbtope_Variable_non_redundant and Lbtope_Confirm dataset. We developed various models on these datasets for discriminating B-cell epitopes from non-epitopes. A web server has been developed for predicting B-cell epitopes using best models developed on these datasets.

## Materials and Methods

We have obtained experimentally validated 49694 B-cell epitopes and 50324 non B-cell epitopes from Immune Epitope Database (IEDB) in Jan 2012 [13]. These epitopes have 2 to 85 amino acids and belong to 3689 antigen sequences. We created five different datasets namely Lbtope_Fixed, Lbtope_Fixed_non_redundant, Lbtope_Variable, Lbtope_Variable_non_redundant, Lbtope_Variable and Lbtope_Confirm from this main dataset. The description of each dataset is as follows:

### 

#### Lbtope_Fixed Dataset

Most of the machine learning techniques commonly used for developing prediction or class discrimination need definite length patterns. Since B-cell epitopes have variable length, we used truncation and extension technique used in previous studies to generate definite length peptides (epitopes & non-epitopes) of 20 residues [8]–[11]. Following procedure has been adopted to generate fixed length epitopes; (i) all epitopes having less than five residues were removed, (ii) epitopes having more than 20 residues were trimmed from both ends to generate epitope of 20 residues from middle, (iii) epitopes containing less than 20 residues have been extended to 20 by adding an equal number of residues at both ends of the epitope, and (iv) finally, identical epitopes were removed. In order to extend an epitope, we mapped it on source antigen from where it has been derived and then we extended its length. In summary, Lbtope_Fixed dataset contains unique 19803 positive patterns or B-cell epitopes and 28329 negative patterns or non B-cell epitopes, where each pattern contains 20-residues. We also removed patterns common in both types of patterns. Our final Lbtope_fixed dataset contains 12063 B-cell epitopes and 20589 non-epitopes (Figure S3).

#### Lbtope_Fixed_non_redundant

Using Lbtope_Fixed dataset, we have created an 80% non-redundant dataset using CD-HIT [14], [15] **.** The redundant dataset contains 7824 B-cell epitopes and 7853 non-epitopes.

#### Lbtope_Variable

First, we removed all epitopes or non-epitopes having less than five residues or more than fifty residues. All epitopes common in B-cell epitopes and non B-cell epitopes were also removed. We found that majority of common epitopes are related to autoimmunity. Our final dataset Lbtope_Variable contains 14876 unique B-cell epitopes and 23321 unique non B-cell epitopes.

#### Lbtope_Variable_non_redundant

We again created an 80% non-redundant Lbtope_Variable dataset using CD-HIT. We obtained 8011 B-cell epitopes and 10868 non-epitopes.

#### Lbtope_Confirm

One of the challenges in creating dataset is its validity, though all epitopes, which we have extracted from IEDB, are experimentally tested. In order to improve the quality of epitopes/non-epitopes, we used only those epitopes/non-epitopes which reported in at least two studies. The final dataset Lbtope_Confirm contains 1042 unique B-cell epitopes and 1795 non B-cell epitopes.

### Input Features

In this study, we generated and used various types of features of peptides that include binary profile or sparse matrix [16], physico-chemical profile (here only four properties tested) [17], dipeptide composition, Chen’s amino acid pair (AAP) propensities [18] and Composition-Transition-Distribution (CTD) profile [14]. All these features were already used in earlier methods (Text S1). Besides these features, we created modified AAP profile, termed as AAP* where instead of multiplication, each dipeptide value was assigned values to each dipeptide from a matrix, making a vector of 19 in place of 400 (Figure S1).

### SVM and Weka Classifiers

In this study, we used SVM_light (http://svmlight.joachims.org/) package for implementing SVM technique. SVM has been used in several biological problems, including functional characterization of proteins [16], [19], [20]. Weka is a tool in which a number of algorithms like Baysian Network, SVMLib, Artificial Neural Network (ANN), Nearest Neighbor (IBk), Random Forest, etc. have been integrated with a user friendly, graphical front end. In weka, performance of all the methods can be compared on the single data set.

### Cross Validation and Performance Measures

Although leave-one out or jackknife test is the best among cross-validation techniques, due to its time-consuming and heavy CPU requirements, n-fold cross validation is the optimum choice [21]. In this study, we have used five-fold cross validation on 90% data and remaining 10% data is used as independent dataset. We calculated sensitivity (Sen), Specificity (Spe), accuracy (Acc) and Matthew’s correlation coefficient (MCC) on an independent dataset. For detail description of these parameters see Ansari *et al* [20].

## Results

### Analysis of B-cell Epitopes

**Figure 1:**
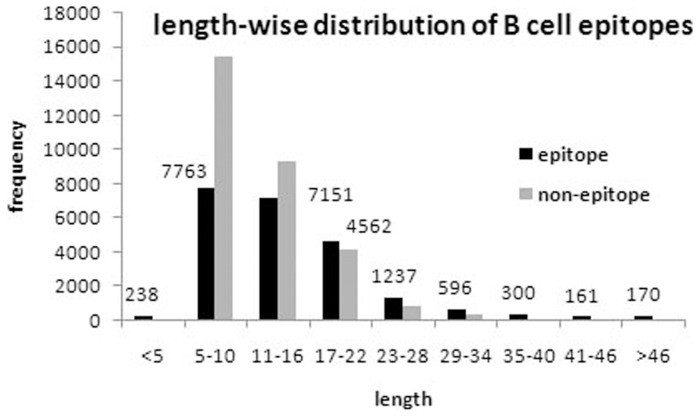
Length-wise distribution of peptides (B-cell epitopes and non-epitopes), we divided peptides in different bins like peptides having a length less than five residues, having residues between 5 to 10 residues.

**Figure 2:**
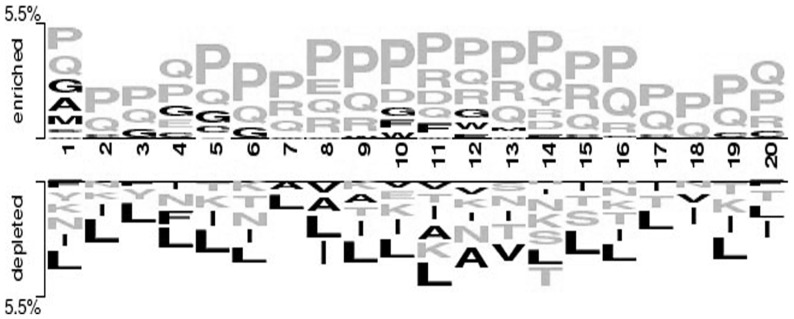
Two-sample logo showing dominance of surface accessible residues in B-cell epitopes. Yellow and black color residues indicate to surface accessible and non-accessible residues respectively.

We analyzed B-cell epitopes to understand their charters tics. First, length wise distribution of B-cell epitopes was computed. As shown in Figure 1, most of the epitopes are in the range of 5–22 amino acid length. In order to understand the preference of residues in B-cell epitopes, we generated two-sample logo plot [22] using 20 mer epitope (upper panel) and non-epitope (lower panel). As shown in Figure 2, there is indeed elevated occurrence of surface accessible and flexible residue in the epitope region as compared to the non-epitope region. In addition, we observed high propensity of Proline and Glycine residue in the epitope region, which might be responsible for the creation of bends or flexibility in the epitope region.

### Performance of Binary Profile Based Models

We developed models for discriminating B-cell epitopes from non B-cell epitopes on Lbtope_Fixed dataset. SVM-based models have been developed using binary profile or sparse profile of patterns, which is represented by a vector length of Wx21 (W is window length, 20 in this study). Sparse matrix contains information for each position and each type of amino acids in the pattern. We achieved accuracy range from 37–67% with MCC of 0.03–0.22 and AUC of 0.65, which is better than random prediction (Table 1; Table S3).

**Table 1:** The performance of SVM models developed on Lbtope_Fixed dataset using various features.

Features/Parameters	Threshold	Sensitivity	Specificity	Accuracy	MCC	AUC
**Binary**	−0.3	67.92	53.10	58.48	0.20	0.65
**Physico-chemical property**	−0.2	58.17	52.81	54.76	0.11	0.58
**Amino acid composition**	−0.3	74.08	79.71	77.67	0.53	0.85
**Composition Transition**	−0.4	67.67	67.33	67.45	0.34	0.72
**Amino Acid Pairs (AAP)**	−0.2	81.75	77.62	79.12	0.58	0.86
AAP*	0	66.67	53.48	58.27	0.19	0.64
**Dipeptide composition**	−0.3	80.5	81.67	81.24	0.61	0.88

These models were developed using 5-fold cross-validation on 90% data and tested on remaining 10% data.

AAP*: Modified AAP where in place of multiplication, simple matrix assignment is used.

### Performance of Models Based on Physico-chemical Properties

It is already known that physico-chemical properties of amino acids are responsible for structural and functional behavior of peptides and proteins. In our study, we have tested few topological properties (Table S2), which were shown to be a good index for B-cell epitope prediction such as relative connectivity, clustering coefficient, closeness and betweenness [23]. We developed SVM models using physico-chemical properties and achieved accuracy in the range of 43–64% with MCC of 0.06–0.13 and AUC of 0.58, which is poorer than models based on binary profile (Table 1; Table S4).

### Performance of Composition Based Models

Besides understanding the positional effect of amino acids, we also computed and compared the overall composition of epitopes and non-epitopes (Figure S2). Similar to two-sample logo analysis, composition analysis revealed that it can be used to discriminate between epitopes and non-epitopes. Therefore, we have applied several distinctive types of models such as simple amino acid and dipeptide composition with different vector size. These models were trained and tested using SVM and IBk. While using SVM, simple amino acid composition performed best among binary and physico-chemical profiles with accuracy of 78%, MCC 0.53 and AUC 0.85. Dipeptide composition model performed better than Chen’s AAP with maximum accuracy of 81%, MCC 0.61 and AUC of 0.88, the highest among single feature models (Table S5, S6, S7, S8, S9). We tested different features of other algorithms implemented in Weka and found that IBk model performed best (results of other algorithms not shown). Dipeptide composition model performed better than AAP profile with accuracy of 81%, MCC 0.61 and AUC 0.86 (Table 2; Table S9).

**Table 2:** The performance of IBk models developed on Lbtope_Fixed dataset using various features.

Features/Parameters	Threshold	Sensitivity	Specificity	Accuracy	MCC	AUC
**Amino acid composition**	0.3	78.5	74.05	75.67	0.51	0.83
**Composition Transition**	0.4	68.17	69.95	69.3	0.37	0.73
**Amino Acid Pairs**	0.4	78.25	81.76	80.48	0.59	0.83
**Dipeptide composition**	0.3	80.33	81.67	81.18	0.61	0.86

These models were developed using 5-fold cross-validation on 90% data and tested on remaining 10% data.

Since only composition-based method can be applied to variable data, we applied amino acid composition, CTD, AAP and dipeptide composition methods on Lbtope_Variable and Lbtope_Confirm datasets. It was observed that dipeptide-based method performed best among other methods with accuracy 75.89, 82.33 and MCC 0.51, 0.64 on Lbtope_Variable, Lbtope_Confirm dataset respectively (Table 3, 4, 5, 6; Table S10, S11, S12, S13, S14, S15, S16, S17). Performance of models was better in case of Lbtope_Confirm data as compare to Lbtope_Variable.

**Table 3:** The performance of SVM models developed on Lbtope_Variable dataset using various features.

Features/Parameters	Threshold	Sensitivity	Specificity	Accuracy	MCC	AUC
**Amino acid composition**	−0.4	68.06	66.31	66.99	0.34	0.73
**Composition Transition**	−0.1	67.72	64.42	65.71	0.31	0.77
**Amino Acid Pairs**	0	76.06	63.87	68.61	0.39	0.72
**Dipeptide composition**	−0.2	75.18	76.34	75.89	0.51	0.83

These models were developed using 5-fold cross-validation on 90% data and tested on remaining 10% data.

**Table 4:** The performance of IBk models developed on Lbtope_Variable dataset using various features.

Features/Parameters	Threshold	Sensitivity	Specificity	Accuracy	MCC	AUC
**Amino acid composition**	0.4	70.41	72.82	71.88	0.42	0.77
**Composition Transition**	0.4	66.38	61.12	63.17	0.27	0.68
**Amino Acid Pairs**	0.3	77.67	76.68	77.07	0.53	0.81
**Dipeptide composition**	0.2	80.90	77.50	78.82	0.57	0.84

These models were developed using 5-fold cross-validation on 90% data and tested on remaining 10% data.

**Table 5:** The performance of SVM models developed on Lbtope_Confirm (epitope tested by at least two studies) dataset using various features.

Features/Parameters	Threshold	Sensitivity	Specificity	Accuracy	MCC	AUC
**Amino acid composition**	0	81.73	73.18	76.33	0.53	0.84
**Composition Transition**	−0.1	76.92	74.86	75.62	0.50	0.82
**Amino Acid Pairs**	0	81.73	73.74	76.68	0.54	0.83
**Dipeptide composition**	−0.3	84.62	81.01	82.33	0.64	0.91

These models were developed using 5-fold cross-validation on 90% data and tested on remaining 10% data.

**Table 6:** The performance of IBk models developed on Lbtope_Confirm (epitope tested by at least two studies) dataset using various features.

Features/Parameters	Threshold	Sensitivity	Specificity	Accuracy	MCC	AUC
**Amino acid composition**	0.3	80.77	77.09	78.45	0.56	0.90
**Composition Transition**	0.3	73.08	73.18	73.14	0.45	0.79
**Amino Acid Pairs**	0.4	87.5	81.56	83.75	0.67	0.85
**Dipeptide composition**	0.4	82.69	87.71	85.87	0.70	0.92

These models were developed using 5-fold cross-validation on 90% data and tested on remaining 10% data.

### Performance on Non-redundant Peptide Dataset

Although we have considered unique epitopes, the redundancy could be expected among them similar to protein sequences. However, it is known that properties of peptide could change with a single amino acid variation. Nevertheless, to have an idea of redundancy and model performance, we created non-redundant dataset corresponding to both Lbtope_Fixed and Lbtope_Variable databases. We found that the number of peptides decreased as expected, but the performance remained significant.

We observed AUC of 0.61, 0.66 and 0.69 for simple amino acid, AAP and dipeptide composition respectively on Lbtope_Fixed_non_redundant dataset (Table S18, S19, S20, S21). We achieved better performance for Lbtope_Variable_non-redundant dataset. The AUC obtained was 0.60, 0.68 and 0.73 for simple amino acid, AAP and dipeptide composition respectively (Table S22, S23, S24, S25). In case of Lbtope_Fixed_non_redundant dataset, AUC sharply decreased from 0.88 to 0.69 and for Lbtope_Variable_non_redundant dataset AUC decreased from 0.83 to 0.73. Again, dipeptide based model performed better than other methods (Table 7–8; Table S18, S19, S20, S21, S22, S23, S24, S25).

**Table 7:** The performance of SVM models developed on Lbtope_Fixed_non_redundant dataset using various features.

Features/Parameters	Threshold	Sensitivity	Specificity	Accuracy	MCC	AUC
**Amino acid composition**	0.0	59.35	57.31	58.33	0.17	0.61
**Composition Transition**	0.0	54.38	57.31	55.85	0.12	0.57
**Amino Acid Pairs**	0.0	65.88	60.57	63.23	0.26	0.66
**Dipeptide composition**	0.0	65.75	63.97	64.86	0.30	0.69

These models were developed using 5-fold cross-validation on 90% data and tested on remaining 10% data.

**Table 8:** The performance of SVM models developed on Lbtope_Variable_non_redundant dataset using various features.

Features/Parameters	Threshold	Sensitivity	Specificity	Accuracy	MCC	AUC
**Amino acid composition**	−0.3	56.41	59.9	58.39	0.16	0.60
**Composition Transition**	−0.6	58.41	66.86	63.19	0.25	0.66
**Amino Acid Pairs**	−0.3	63.51	62.95	63.19	0.26	0.68
**Dipeptide composition**	−0.1	66	67.24	66.7	0.33	0.73

These models were developed using 5-fold cross-validation on 90% data and tested on remaining 10% data.

### Benchmarking with Existing Methods

It is important to compare the newly developed algorithm with existing algorithms, which requires testing of all methods on same dataset. Unfortunately, our dataset is different than datasets used in previous studies. Thus one to one comparison is not feasible. In order to understand differences and similarities in our and existing models, we tested our models on datasets used in earlier methods. Similarly, we tested previously developed methods on our datasets. It was observed that earlier models failed on datasets used in this study, and our models failed on existing datasets (Table S26, S27, S28, S29, S30, S31, S32). Authors of ABCpred achieved sensitivity 57.14 and specificity 71.57 at the default threshold on their ABCpred dataset (Table 9). We assessed the performance of ABCpred at the default threshold on dataset Lbtope_Fixed used in this study and achieved sensitivity 54.55 and specificity 49.54 [11]. It was observed that sensitivity of ABCpred decreased slightly from 57.14 to 54.55 but specificity decreased drastically from 71.57 to 49.54 (Table 9). It suggested that ABCpred performance on B-cell epitopes decreased slightly but failed on non B-cell epitopes used in this study. We also evaluated models developed in this study on ABCpred and observed similar results. Models developed in this study failed on non B-cell epitopes (random peptides generated from proteins) used in ABCpred dataset (Table 9; Table S30, S31).

**Table 9:** The performance of our method LBtope on ABCpred dataset and performance of ABCpred on dataset Lbtope_Fixed (fixed length patterns used in this study).

Performance on Datasets/Parameters	Sensitivity	Specificity	Accuracy	MCC
**Performance of ABCpred on Lbtope_Fixed dataset**	54.55	49.54	51.39	0.04
**ABCpred performance on ABCpred dataset**	57.14	71.57	64.36	0.29
**Performance of LBtope model trained on Lbtope_Fixed on ABCpred dataset**	70	33.71	57.9	0.04

Similarly, we evaluated existing methods (BCPred and Chen’s method) on datasets used in this study. It was observed that these methods performed reasonably well on B-cell epitopes but failed on non B-cell epitopes (Table 10; Table S26, S27, S28, S29) [9], [10]. We also evaluated the performance of our dipeptide-based models on datasets used in previous studies (Table 10; Table S32) [14]. We observed similar trend, our models failed on negative patterns/example (random peptides used as non B-cell epitopes).

**Table 10:** The performance of BCPred, Chen on Lbtope_fixed and, Lbtope_variable dataset and performance of LBtope models on datasets used in previous studies.

Performance of models/Parameters	Sensitivity	Specificity	Accuracy	MCC
**BCPred on Lbtope_Fixed dataset**	58.87	50.47	53.57	0.09
**LBtope model on BCPred dataset (trained on Lbtope_Fixed dataset)**	69.33	33.81	51.57	0.03
**Chen’s AAP model on Lbtope_Fixed dataset**	62.1	43.26	50.22	0.05
**LBtope model on Chen’s dataset (trained on Lbtope_Fixed dataset)**	70.76	35.89	53.33	0.07
**LBtope model on BCPred dataset (trained on Lbtope_Variable dataset)**	74.49	40.64	57.56	0.16
**LBtope model on BCPred flexible dataset (trained on Lbtope_Confirm dataset)**	78.09	27.23	52.66	0.06
**Five-fold cross validation Lbtope_confirm dataset**	84.86	81.37	82.65	0.65
**Five-fold cross validation Lbtope_positive_fbcpred_negative dataset**	85.82	85.65	85.74	0.71

Performance of LBtope models on Lbtope_Confirm and Lbtope_positive_fbcpred_negative datasets.

It can be suggested that existing models/methods perform reasonably fine on our B-cell epitopes, but failed on non B-cell epitopes. Similarly, our models failed on random peptides used in previous studies as non B-cell epitopes. This could be due to fact that our negative dataset comprised of experimentally verified non B-cell epitopes where as negative datasets of existing methods consist of random peptides generated from proteins.

To know the effect of using experimental proved non B-cell epitope instead of random peptides from Swiss-Prot in development of model. We created another dataset Lbtope-positive-fbcpred-negative, in which instead of experimental non B-cell epitopes, we used random peptides from FBCPred dataset as non B-cell epitopes. Next, we performed a five-fold cross validation on the above- dataset and obtained 85% sensitivity with 0.71 MCC. On the other hand, Lbtope_Confirm has achieved 81% sensitivity with 0.65 MCC, a bit poorer than Lbtope-positive-fbcpred-negative (Table 10; Table S33–S34). Taken together all this results, it can be concluded that using experimental B-cell epitopes, the method can perform as good as using random peptides. In summary, performance of models depends upon the dataset used for training.

### Implementation

In order to provide prediction service to scientific community, we have developed a user-friendly web server based on the model developed in this study. The server is developed using PHP 5.2.9, HTML and JavaScript as the front end and installed on a Red Hat Enterprise Linux 6 server environment. The server takes antigen primary amino acid sequence (s) in ‘FASTA’ format, generates 20 amino acids overlapping peptides for Lbtope_Fixed dataset model, 5–30 amino acids overlapping peptides for variable datasets model and predicts the linear epitopes. The non-redundant model is also implemented in case of very high specificity. The output is antigen sequence (s) mapped with B-cell epitopes with a probability scale of 20–80%. A higher score implies higher probability of peptide to be B-cell epitope. We have developed separate dedicated pages for antigen and peptide submission to avoid any complexity. In addition, we have developed a peptide mutation tool, which creates all possible single point mutations in given peptide and calculates the probability score based on the algorithm and also predicts the other properties. Using the mutation tool, user can design better epitopes or even choose fewer epitopic peptides for the de-immunization of therapeutic proteins. The web server is freely available at http://crdd.osdd.net/raghava/lbtope/.

## Discussion

Epitope mapping is no doubt a very useful procedure, which has vast applications in the area of therapy and diagnostics. Experimental methods do exist, but they require time, resources and cannot handle the pace with which biological data is generated. Therefore, computer algorithms have been developed over the decades to predict the B- and T-cell epitopes from antigen sequence or structure if available. It’s observed that linear B-cell epitope prediction is more challenging than other epitope types like conformational B-cell or T-cell epitopes. This might be due to the reason that linear B-cell epitope posse’s variable length from 2–85 amino acids as compared to the almost fixed length core of the T-cell epitopes. This variability imposes several obstacles for algorithm developers. Besides variability, all the methods to date have been developed on very small data set with negatives examples obtained from randomly chosen UniProt peptides or same antigens, which are not experimentally validated. In the present study, for the first time we have used experimentally verified B-cell and non B-cell epitopes from IEDB database, which are much more in the number and rationally, created to previous methods. We created the 20 mer epitopes using corresponding ‘truncation-extension’ methodology and similar length, which were used in earlier methods. By using simple composition technique in combination with SVM and Weka implemented IBk; we came up with an algorithm, which is as good as existing tools. Performance of LBtope models decreased on non-redundant datasets, still performance remained as good as existing methods. It is also observed that model developed on Lbtope_Confirm dataset performed better than the models developed on Lbtope_Variable dataset. We have compared the performance of LBtope models on Lbtope and existing datasets. LBtope performs poor on negative dataset of existing methods, and they also performed poor on our negative dataset. It is because our negative dataset is experimentally verified B-cell epitope, whereas existing method, negative dataset were randomly generated from UniProt. We have implemented the algorithm in the form of a user-friendly web server: LBtope. The user can create the mutants of each peptide and test its epitopic or other desired probability using our server’s mutant tool. We hope that present model will aid the researchers in the field of linear B-cell epitope prediction.

## Supporting Information

Figure S1 Diagram showing calculation of Dipeptide composition, AAP and modified AAP (AAP*) from patterns.
(TIF)

Figure S2 Diagram showing % composition of B-cell epitopes and non-epitopes (LBtope data; 20 mers).
(TIF)

Figure S3 Flowchart showing preparation of LBtope dataset from IEDB database.
(TIF)

Table S1 Datasets used so far in the linear B-cell epitope prediction.
(DOC)

Table S2 Amino acid indices as obtained from Huang et al 2007.
(DOC)

Table S3 The performance of SVM models developed on Lbtope_Fixed dataset using binary profile. These models were developed using 5-fold cross-validation on 90% data and tested on remaining 10% data.
(DOC)

Table S4 The performance of SVM models developed on Lbtope_Fixed dataset using Physico-chemical property (4 R indices). These models were developed using 5-fold cross-validation on 90% data and tested on remaining 10% data.
(DOC)

Table S5 The performance of SVM/IBK models developed on Lbtope_Fixed dataset using Amino acid composition. These models were developed using 5-fold cross-validation on 90% data and tested on remaining 10% data.
(DOC)

Table S6 The performance of SVM/IBK models developed on Lbtope_Fixed dataset using Composition Transition. These models were developed using 5-fold cross-validation on 90% data and tested on remaining 10% data.
(DOC)

Table S7 The performance of SVM/IBK models developed on Lbtope_Fixed dataset using AAP profile. These models were developed using 5-fold cross-validation on 90% data and tested on remaining 10% data.
(DOC)

Table S8 The performance of SVM/IBK models developed on Lbtope_Fixed dataset using AAA profile. These models were developed using 5-fold cross-validation on 90% data and tested on remaining 10% data.
(DOC)

Table S9 The performance of SVM/IBK models developed on Lbtope_Fixed dataset using Dipeptide composition. These models were developed using 5-fold cross-validation on 90% data and tested on remaining 10% data.
(DOC)

Table S10 The performance of SVM/IBK models developed on Lbtope_Variable dataset using Amino acid composition. These models were developed using 5-fold cross-validation on 90% data and tested on remaining 10% data.
(DOC)

Table S11 The performance of SVM/IBK models developed on Lbtope_Variable dataset using Composition Transition. These models were developed using 5-fold cross-validation on 90% data and tested on remaining 10% data.
(DOC)

Table S12 The performance of SVM/IBK models developed on Lbtope_Variable dataset using AAP profile. These models were developed using 5-fold cross-validation on 90% data and tested on remaining 10% data.
(DOC)

Table S13 The performance of SVM/IBK models developed on Lbtope_Variable dataset using Dipeptide composition. These models were developed using 5-fold cross-validation on 90% data and tested on remaining 10% data.
(DOC)

Table S14 The performance of SVM/IBK models developed on Lbtope_Confirm (epitope tested by at least two studies) dataset using Amino acid composition. These models were developed using 5-fold cross-validation on 90% data and tested on remaining 10% data.
(DOC)

Table S15 The performance of SVM/IBK models developed on Lbtope_Confirm (epitope tested by at least two studies) dataset using Composition Transition. These models were developed using 5-fold cross-validation on 90% data and tested on remaining 10% data.
(DOC)

Table S16 The performance of SVM/IBK models developed on Lbtope_Confirm (epitope tested by at least two studies) dataset using AAP profile. These models were developed using 5-fold cross-validation on 90% data and tested on remaining 10% data.
(DOC)

Table S17 The performance of SVM/IBK models developed on Lbtope_Confirm (epitope tested by at least two studies) dataset using Dipeptide composition. These models were developed using 5-fold cross-validation on 90% data and tested on remaining 10% data.
(DOC)

Table S18 The performance of SVM/IBK models developed on Lbtope_Fixed_non_redundant dataset using amino acid composition. These models were developed using 5-fold cross-validation on 90% data and tested on remaining 10% data.
(DOC)

Table S19 The performance of SVM/IBK models developed on Lbtope_Fixed_non_redundant dataset using composition-transition. These models were developed using 5-fold cross-validation on 90% data and tested on remaining 10% data.
(DOC)

Table S20 The performance of SVM/IBK models developed on Lbtope_Fixed_non_redundant dataset using composition-transition. These models were developed using 5-fold cross-validation on 90% data and tested on remaining 10% data.
(DOC)

Table S21 The performance of SVM/IBK models developed on Lbtope_Fixed_non_redundant dataset using dipeptide composition. These models were developed using 5-fold cross-validation on 90% data and tested on remaining 10% data.
(DOC)

Table S22 The performance of SVM/IBK model developed on Lbtope_Variable_non_redundant dataset using amino acid composition. These models were developed using 5-fold cross-validation on 90% data and tested on remaining 10%.
(DOC)

Table S23 The performance of SVM/IBK models developed on Lbtope_Variable_non_redundant dataset using composition-transition. These models were developed using 5-fold cross-validation on 90% data and tested on remaining 10% data.
(DOC)

Table S24 The performance of SVM/IBK models developed on Lbtope_Variable_non_redundant dataset using composition-transition. These models were developed using 5-fold cross-validation on 90% data and tested on remaining 10% data.
(DOC)

Table S25 The performance of SVM/IBK models developed on Lbtope_Variable_non_redundant dataset using dipeptide composition. These models were developed using 5-fold cross-validation on 90% data and tested on remaining 10% data.
(DOC)

Table S26 The performance of Chen’s AAP model on Lbtope_Fixed data.
(DOC)

Table S27 Performance of BCPred model (20 mer) on Lbtope_Fixed dataset.
(DOC)

Table S28 The performance of SVM models developed on Lbtope_Fixed dataset tested on Chen dataset.
(DOC)

Table S29 The performance of SVM models developed on Lbtope_Fixed dataset tested on BCPred dataset.
(DOC)

Table S30 Performance of ABCPred model (20 mer) on Lbtope_Fixed dataset.
(DOC)

Table S31 The performance of SVM models developed on Lbtope_Fixed and tested on ABCPred dataset.
(DOC)

Table S32 The performance of SVM models developed on Lbtope_Variable and tested on bcpred variable data.
(DOC)

Table S33 SVM results of five-fold cross validation on Lbtope_Confirm (epitope tested by at least two studies) dataset using dipeptide composition.
(DOC)

Table S34 SVM results of five-fold cross validation on Lbtope_positive_fbcpred_negative (B-cell epitope (positive) from Lbtope_Confirm dataset and random peptides (negative) from fbcpred) using dipeptide composition.
(DOC)

Text S1 Different B-cell epitope prediction techniques already used in earlier methods.
(DOC)
