# Supplementary figures and images for: Improved Method for Linear B-Cell Epitope Prediction Using Antigen’s Primary Sequence
Source: PLoS One. 2013 May 7;8(5):e62216. doi: 10.1371/journal.pone.0062216 (PMC3646881; doi:10.1371/journal.pone.0062216)

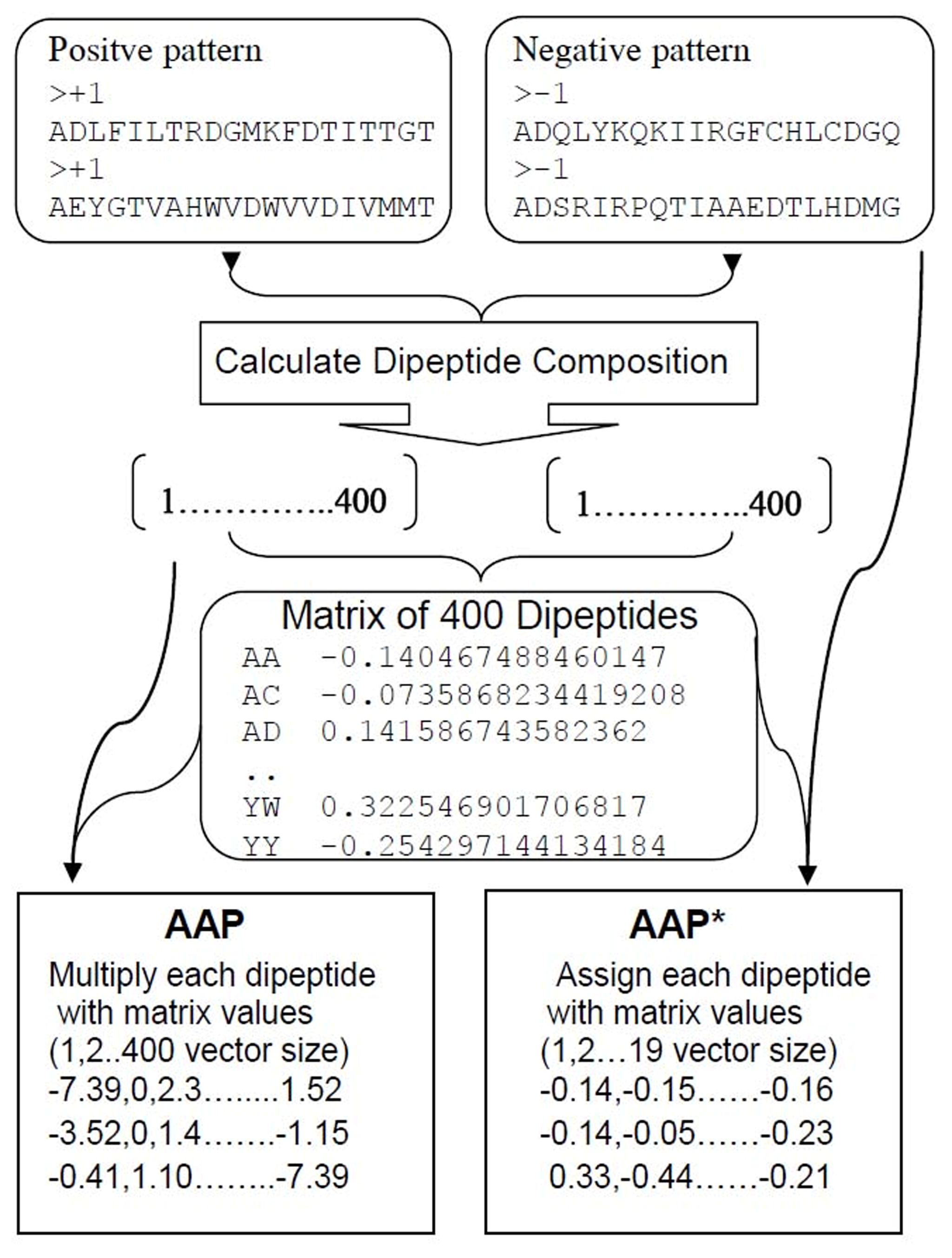

Supplement: Figure S1 — Diagram showing calculation of Dipeptide composition, AAP and modified AAP (AAP*) from patterns. (TIF) [file pone.0062216.s001.tif]

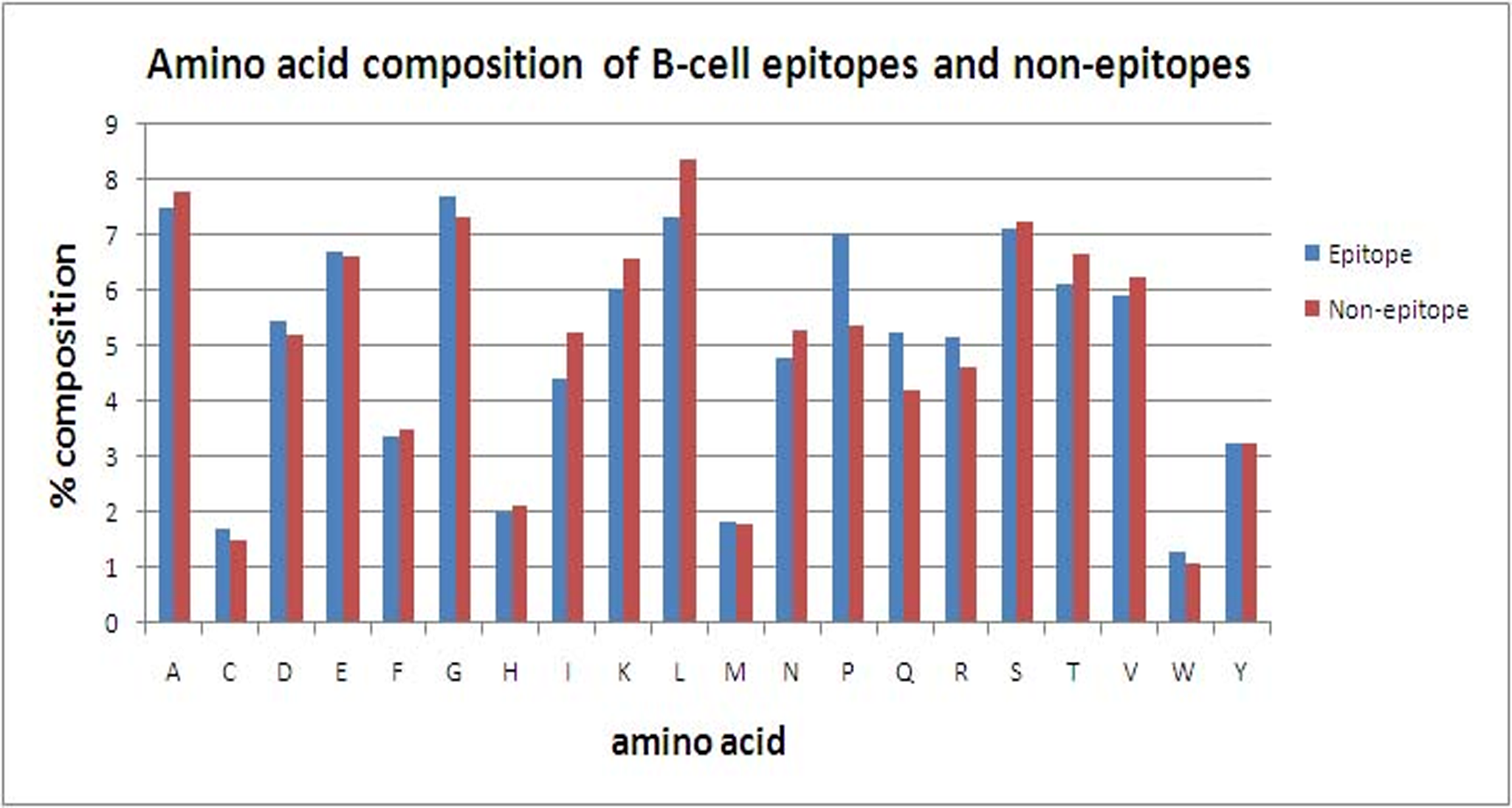

Supplement: Figure S2 — Diagram showing % composition of B-cell epitopes and non-epitopes (LBtope data; 20 mers). (TIF) [file pone.0062216.s002.tif]

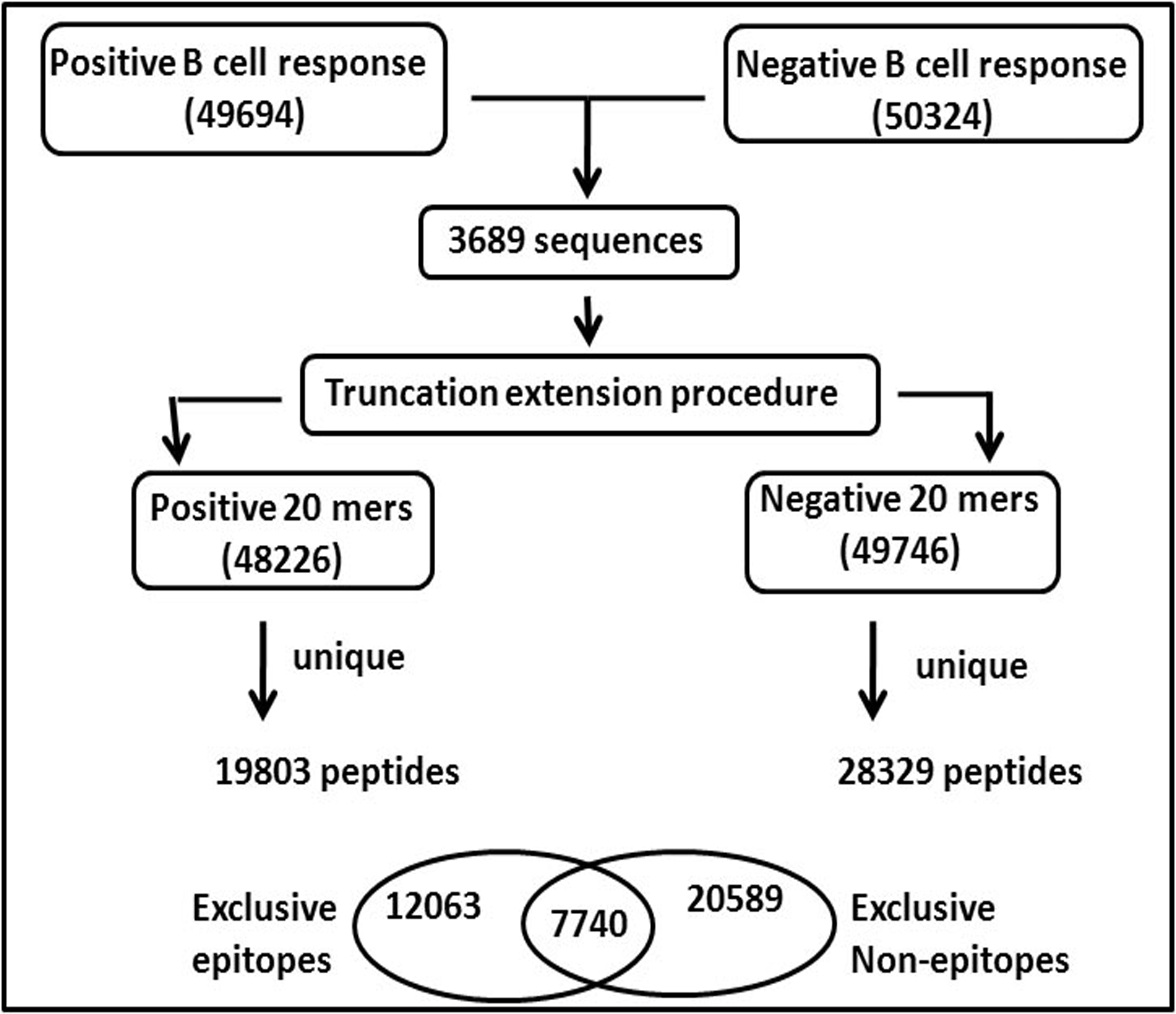

Supplement: Figure S3 — Flowchart showing preparation of LBtope dataset from IEDB database. (TIF) [file pone.0062216.s003.tif]
