# Supplementary material for: Improved Method for Linear B-Cell Epitope Prediction Using Antigen’s Primary Sequence
Source: PLoS One. 2013 May 7;8(5):e62216. doi: 10.1371/journal.pone.0062216 (PMC3646881; doi:10.1371/journal.pone.0062216)
Supplement: Table S1 — Datasets used so far in the linear B-cell epitope prediction. (DOC) [file pone.0062216.s004.doc]

Table S1. Datasets used so far in the linear B-cell epitope prediction

| **Dataset** | **Epitope/positive** | **Non-epitope/**  **negative** | **Redundancy** | **Reference** |
| --- | --- | --- | --- | --- |
| ABCPred/Bcipep | 700 | 700 (random peptides) | Redundant | (Saha and Raghava, 2006) |
| Chen | 872 | 872 (random peptides) | Redundant | (Chen et al., 2007) |
| BCPred (fixed) | 701 | 701(random peptides) | 80% non-redundant | (El-Manzalawy et al., 2008) |
| BCPred (variable) | 1223 | 1223(random peptides) | Non redundant | (El-Manzalawy et al., 2008) |
