# Supplementary material for: Improved Method for Linear B-Cell Epitope Prediction Using Antigen’s Primary Sequence
Source: PLoS One. 2013 May 7;8(5):e62216. doi: 10.1371/journal.pone.0062216 (PMC3646881; doi:10.1371/journal.pone.0062216)
Supplement: Table S2 — Amino acid indices as obtained from Huang et al 2007. (DOC) [file pone.0062216.s005.doc]

**Table S2. Amino acid indices as obtained from Huang et al 2007**

| **Prop.*** | **A** | **C** | **D** | **E** | **F** | **G** | **H** | **I** | **K** | **L** | **M** | **N** | **P** | **Q** | **R** | **S** | **T** | **V** | **W** | **Y** |
| --- | --- | --- | --- | --- | --- | --- | --- | --- | --- | --- | --- | --- | --- | --- | --- | --- | --- | --- | --- | --- |
| **Rk** | 1.05 | 1.17 | 0.88 | 0.85 | 1.07 | 0.99 | 0.99 | 1.11 | 0.88 | 1.07 | 1.04 | 0.93 | 0.92 | 0.93 | 0.94 | 0.96 | 0.99 | 1.12 | 1.05 | 1.05 |
| **Rc** | 0.99 | 0.89 | 1.11 | 1.13 | 0.92 | 1.08 | 1 | 0.89 | 1.1 | 0.92 | 0.95 | 1.07 | 1.01 | 1.06 | 1.04 | 1.05 | 1.01 | 0.9 | 0.93 | 0.94 |
| **Ro** | 1.00 | 1.13 | 0.95 | 0.95 | 1.03 | 0.99 | 1.01 | 1.04 | 0.96 | 1.02 | 1.02 | 0.96 | 0.96 | 0.97 | 0.98 | 0.98 | 0.99 | 1.04 | 1.01 | 1.02 |
| **Rb** | 0.96 | 1.6 | 0.63 | 0.61 | 1.31 | 0.77 | 1.03 | 1.43 | 0.61 | 1.3 | 1.24 | 0.72 | 0.83 | 0.73 | 0.82 | 0.8 | 0.9 | 1.35 | 1.2 | 1.16 |

*Prop. - Property, Relative connectivity (Rk), Relative clustering coefficient (Rc), Relative closeness (Ro), Relative betweenness (Rb)
