# Supplementary material for: Improved Method for Linear B-Cell Epitope Prediction Using Antigen’s Primary Sequence
Source: PLoS One. 2013 May 7;8(5):e62216. doi: 10.1371/journal.pone.0062216 (PMC3646881; doi:10.1371/journal.pone.0062216)
Supplement: Table S3 — The performance of SVM models developed on Lbtope_Fixed dataset using binary profile. These models were developed using 5-fold cross-validation on 90% data and tested on remaining 10% data. (DOC) [file pone.0062216.s006.doc]

**Table S3. The performance of SVM models developed on Lbtope_Fixed dataset using binary profile. These models were developed using 5-fold cross-validation on 90% data and tested on remaining 10% data.**

| **Thres** | **TP** | **FP** | **TN** | **FN** | **Sen** | **Spec** | **Accuracy** | **MCC** |
| --- | --- | --- | --- | --- | --- | --- | --- | --- |
| -1 | 1196 | 2092 | 8 | 4 | 99.67 | 0.38 | 36.48 | 0.00 |
| -0.9 | 1191 | 2074 | 26 | 9 | 99.25 | 1.24 | 36.88 | 0.02 |
| -0.8 | 1181 | 2015 | 85 | 19 | 98.42 | 4.05 | 38.36 | 0.07 |
| -0.7 | 1157 | 1941 | 159 | 43 | 96.42 | 7.57 | 39.88 | 0.08 |
| -0.6 | 1120 | 1802 | 298 | 80 | 93.33 | 14.19 | 42.97 | 0.11 |
| -0.5 | 1052 | 1592 | 508 | 148 | 87.67 | 24.19 | 47.27 | 0.14 |
| -0.4 | 951 | 1283 | 817 | 249 | 79.25 | 38.90 | 53.58 | 0.19 |
| -0.3 | 815 | 985 | 1115 | 385 | 67.92 | 53.10 | 58.48 | 0.20 |
| -0.2 | 665 | 720 | 1380 | 535 | 55.42 | 65.71 | 61.97 | 0.21 |
| -0.1 | 516 | 471 | 1629 | 684 | 43.00 | 77.57 | 65.00 | 0.22 |
| 0 | 373 | 292 | 1808 | 827 | 31.08 | 86.10 | 66.09 | 0.21 |
| 0.1 | 281 | 175 | 1925 | 919 | 23.42 | 91.67 | 66.85 | 0.21 |
| 0.2 | 218 | 102 | 1998 | 982 | 18.17 | 95.14 | 67.15 | 0.22 |
| 0.3 | 176 | 70 | 2030 | 1024 | 14.67 | 96.67 | 66.85 | 0.21 |
| 0.4 | 153 | 47 | 2053 | 1047 | 12.75 | 97.76 | 66.85 | 0.21 |
| 0.5 | 127 | 37 | 2063 | 1073 | 10.58 | 98.24 | 66.36 | 0.20 |
| 0.6 | 106 | 26 | 2074 | 1094 | 8.83 | 98.76 | 66.06 | 0.19 |
| 0.7 | 84 | 17 | 2083 | 1116 | 7.00 | 99.19 | 65.67 | 0.17 |
| 0.8 | 48 | 7 | 2093 | 1152 | 4.00 | 99.67 | 64.88 | 0.14 |
| 0.9 | 29 | 1 | 2099 | 1171 | 2.42 | 99.95 | 64.48 | 0.12 |
| 1 | 23 | 0 | 2100 | 1177 | 1.92 | 100.00 | 64.33 | 0.11 |
