# Supplementary material for: Improved Method for Linear B-Cell Epitope Prediction Using Antigen’s Primary Sequence
Source: PLoS One. 2013 May 7;8(5):e62216. doi: 10.1371/journal.pone.0062216 (PMC3646881; doi:10.1371/journal.pone.0062216)
Supplement: Table S4 — The performance of SVM models developed on Lbtope_Fixed dataset using Physico-chemical property (4 R indices). These models were developed using 5-fold cross-validation on 90% data and tested on remaining 10% data. (DOC) [file pone.0062216.s007.doc]

**Table S4. The performance of SVM models developed on Lbtope_Fixed dataset using Physico-chemical property (4 R indices). These models were developed using 5-fold cross-validation on 90% data and tested on remaining 10% data.**

| **Thres** | **TP** | **FP** | **TN** | **FN** | **Sen** | **Spec** | **Accuracy** | **MCC** |
| --- | --- | --- | --- | --- | --- | --- | --- | --- |
| -1 | 1074 | 1777 | 323 | 126 | 89.5 | 15.38 | 42.33 | 0.07 |
| -0.9 | 1039 | 1692 | 408 | 161 | 86.58 | 19.43 | 43.85 | 0.08 |
| -0.8 | 997 | 1605 | 495 | 203 | 83.08 | 23.57 | 45.21 | 0.08 |
| -0.7 | 950 | 1515 | 585 | 250 | 79.17 | 27.86 | 46.52 | 0.08 |
| -0.6 | 902 | 1423 | 677 | 298 | 75.17 | 32.24 | 47.85 | 0.08 |
| -0.5 | 852 | 1314 | 786 | 348 | 71 | 37.43 | 49.64 | 0.09 |
| -0.4 | 805 | 1222 | 878 | 395 | 67.08 | 41.81 | 51 | 0.09 |
| -0.3 | 755 | 1115 | 985 | 445 | 62.92 | 46.9 | 52.73 | 0.1 |
| -0.2 | 698 | 991 | 1109 | 502 | 58.17 | 52.81 | 54.76 | 0.11 |
| -0.1 | 649 | 874 | 1226 | 551 | 54.08 | 58.38 | 56.82 | 0.12 |
| 0 | 598 | 765 | 1335 | 602 | 49.83 | 63.57 | 58.58 | 0.13 |
| 0.1 | 525 | 652 | 1448 | 675 | 43.75 | 68.95 | 59.79 | 0.13 |
| 0.2 | 479 | 575 | 1525 | 721 | 39.92 | 72.62 | 60.73 | 0.13 |
| 0.3 | 442 | 482 | 1618 | 758 | 36.83 | 77.05 | 62.42 | 0.15 |
| 0.4 | 396 | 420 | 1680 | 804 | 33 | 80 | 62.91 | 0.14 |
| 0.5 | 344 | 353 | 1747 | 856 | 28.67 | 83.19 | 63.36 | 0.14 |
| 0.6 | 297 | 295 | 1805 | 903 | 24.75 | 85.95 | 63.7 | 0.13 |
| 0.7 | 260 | 244 | 1856 | 940 | 21.67 | 88.38 | 64.12 | 0.13 |
| 0.8 | 220 | 200 | 1900 | 980 | 18.33 | 90.48 | 64.24 | 0.13 |
| 0.9 | 172 | 147 | 1953 | 1028 | 14.33 | 93 | 64.39 | 0.12 |
| 1 | 130 | 108 | 1992 | 1070 | 10.83 | 94.86 | 64.3 | 0.11 |
