# Supplementary material for: Improved Method for Linear B-Cell Epitope Prediction Using Antigen’s Primary Sequence
Source: PLoS One. 2013 May 7;8(5):e62216. doi: 10.1371/journal.pone.0062216 (PMC3646881; doi:10.1371/journal.pone.0062216)
Supplement: Table S5 — The performance of SVM/IBK models developed on Lbtope_Fixed dataset using Amino acid composition. These models were developed using 5-fold cross-validation on 90% data and tested on remaining 10% data. (DOC) [file pone.0062216.s008.doc]

**Table S5. The performance of SVM/IBK models developed on Lbtope_Fixed dataset using Amino acid composition. These models were developed using 5-fold cross-validation on 90% data and tested on remaining 10% data.**

| **SVM** | | | | | | | | |
| --- | --- | --- | --- | --- | --- | --- | --- | --- |
| **Thres** | **TP** | **FP** | **TN** | **FN** | **Sen** | **Spec** | **Accuracy** | **MCC** |
| -1 | 1184 | 1754 | 346 | 16 | 98.67 | 16.48 | 46.36 | 0.23 |
| -0.9 | 1170 | 1479 | 621 | 30 | 97.5 | 29.57 | 54.27 | 0.33 |
| -0.8 | 1152 | 1229 | 871 | 48 | 96 | 41.48 | 61.3 | 0.4 |
| -0.7 | 1127 | 1015 | 1085 | 73 | 93.92 | 51.67 | 67.03 | 0.46 |
| -0.6 | 1087 | 827 | 1273 | 113 | 90.58 | 60.62 | 71.52 | 0.5 |
| -0.5 | 1031 | 679 | 1421 | 169 | 85.92 | 67.67 | 74.3 | 0.52 |
| -0.4 | 960 | 548 | 1552 | 240 | 80 | 73.9 | 76.12 | 0.52 |
| -0.3 | 889 | 426 | 1674 | 311 | 74.08 | 79.71 | 77.67 | 0.53 |
| -0.2 | 805 | 321 | 1779 | 395 | 67.08 | 84.71 | 78.3 | 0.53 |
| -0.1 | 699 | 232 | 1868 | 501 | 58.25 | 88.95 | 77.79 | 0.5 |
| 0 | 623 | 173 | 1927 | 577 | 51.92 | 91.76 | 77.27 | 0.49 |
| 0.1 | 541 | 129 | 1971 | 659 | 45.08 | 93.86 | 76.12 | 0.47 |
| 0.2 | 471 | 98 | 2002 | 729 | 39.25 | 95.33 | 74.94 | 0.44 |
| 0.3 | 408 | 75 | 2025 | 792 | 34 | 96.43 | 73.73 | 0.41 |
| 0.4 | 349 | 55 | 2045 | 851 | 29.08 | 97.38 | 72.55 | 0.39 |
| 0.5 | 289 | 41 | 2059 | 911 | 24.08 | 98.05 | 71.15 | 0.35 |
| 0.6 | 239 | 30 | 2070 | 961 | 19.92 | 98.57 | 69.97 | 0.33 |
| 0.7 | 184 | 16 | 2084 | 1016 | 15.33 | 99.24 | 68.73 | 0.29 |
| 0.8 | 142 | 12 | 2088 | 1058 | 11.83 | 99.43 | 67.58 | 0.26 |
| 0.9 | 104 | 5 | 2095 | 1096 | 8.67 | 99.76 | 66.64 | 0.23 |
| 1 | 58 | 2 | 2098 | 1142 | 4.83 | 99.9 | 65.33 | 0.17 |
| IBK | | | | | | | | |
| 0 | 1200 | 2100 | 0 | 0 | 100 | 0 | 36.36 | 0 |
| 0.1 | 1072 | 764 | 1336 | 128 | 89.33 | 63.62 | 72.97 | 0.51 |
| 0.2 | 1035 | 697 | 1403 | 165 | 86.25 | 66.81 | 73.88 | 0.51 |
| 0.3 | 942 | 545 | 1555 | 258 | 78.5 | 74.05 | 75.67 | 0.51 |
| 0.4 | 825 | 393 | 1707 | 375 | 68.75 | 81.29 | 76.73 | 0.5 |
| 0.5 | 734 | 313 | 1787 | 466 | 61.17 | 85.1 | 76.39 | 0.48 |
| 0.6 | 562 | 166 | 1934 | 638 | 46.83 | 92.1 | 75.64 | 0.45 |
| 0.7 | 473 | 106 | 1994 | 727 | 39.42 | 94.95 | 74.76 | 0.43 |
| 0.8 | 420 | 80 | 2020 | 780 | 35 | 96.19 | 73.94 | 0.42 |
| 0.9 | 377 | 65 | 2035 | 823 | 31.42 | 96.9 | 73.09 | 0.4 |
| 1 | 375 | 63 | 2037 | 825 | 31.25 | 97 | 73.09 | 0.4 |
