# Supplementary material for: Improved Method for Linear B-Cell Epitope Prediction Using Antigen’s Primary Sequence
Source: PLoS One. 2013 May 7;8(5):e62216. doi: 10.1371/journal.pone.0062216 (PMC3646881; doi:10.1371/journal.pone.0062216)
Supplement: Table S6 — The performance of SVM/IBK models developed on Lbtope_Fixed dataset using Composition Transition. These models were developed using 5-fold cross-validation on 90% data and tested on remaining 10% data. (DOC) [file pone.0062216.s009.doc]

**Table S6. The performance of SVM/IBK models developed on Lbtope_Fixed dataset using Composition Transition. These models were developed using 5-fold cross-validation on 90% data and tested on remaining 10% data.**

| **SVM** | | | | | | | | |
| --- | --- | --- | --- | --- | --- | --- | --- | --- |
| **Thres** | **TP** | **FP** | **TN** | **FN** | **Sen** | **Spec** | **Accuracy** | **MCC** |
| -1 | 1084 | 1512 | 588 | 116 | 90.33 | 28 | 50.67 | 0.22 |
| -0.9 | 1047 | 1367 | 733 | 153 | 87.25 | 34.9 | 53.94 | 0.24 |
| -0.8 | 1013 | 1234 | 866 | 187 | 84.42 | 41.24 | 56.94 | 0.26 |
| -0.7 | 970 | 1092 | 1008 | 230 | 80.83 | 48 | 59.94 | 0.29 |
| -0.6 | 927 | 965 | 1135 | 273 | 77.25 | 54.05 | 62.48 | 0.30 |
| -0.5 | 877 | 819 | 1281 | 323 | 73.08 | 61 | 65.39 | 0.33 |
| -0.4 | 812 | 686 | 1414 | 388 | 67.67 | 67.33 | 67.45 | 0.34 |
| -0.3 | 760 | 596 | 1504 | 440 | 63.33 | 71.62 | 68.61 | 0.34 |
| -0.2 | 679 | 501 | 1599 | 521 | 56.58 | 76.14 | 69.03 | 0.33 |
| -0.1 | 612 | 419 | 1681 | 588 | 51 | 80.05 | 69.48 | 0.32 |
| 0 | 555 | 320 | 1780 | 645 | 46.25 | 84.76 | 70.76 | 0.34 |
| 0.1 | 497 | 273 | 1827 | 703 | 41.42 | 87 | 70.42 | 0.32 |
| 0.2 | 448 | 216 | 1884 | 752 | 37.33 | 89.71 | 70.67 | 0.32 |
| 0.3 | 408 | 175 | 1925 | 792 | 34 | 91.67 | 70.7 | 0.32 |
| 0.4 | 353 | 146 | 1954 | 847 | 29.42 | 93.05 | 69.91 | 0.3 |
| 0.5 | 311 | 123 | 1977 | 889 | 25.92 | 94.14 | 69.33 | 0.29 |
| 0.6 | 255 | 96 | 2004 | 945 | 21.25 | 95.43 | 68.45 | 0.26 |
| 0.7 | 210 | 70 | 2030 | 990 | 17.5 | 96.67 | 67.88 | 0.24 |
| 0.8 | 171 | 55 | 2045 | 1029 | 14.25 | 97.38 | 67.15 | 0.22 |
| 0.9 | 149 | 39 | 2061 | 1051 | 12.42 | 98.14 | 66.97 | 0.22 |
| 1 | 121 | 28 | 2072 | 1079 | 10.08 | 98.67 | 66.45 | 0.2 |
| IBK | | | | | | | | |
| 0 | 1200 | 2100 | 0 | 0 | 100 | 0 | 36.36 | 0 |
| 0.1 | 893 | 768 | 1332 | 307 | 74.42 | 63.43 | 67.42 | 0.36 |
| 0.2 | 889 | 761 | 1339 | 311 | 74.08 | 63.76 | 67.52 | 0.36 |
| 0.3 | 877 | 730 | 1370 | 323 | 73.08 | 65.24 | 68.09 | 0.37 |
| 0.4 | 818 | 631 | 1469 | 382 | 68.17 | 69.95 | 69.3 | 0.37 |
| 0.5 | 662 | 412 | 1688 | 538 | 55.17 | 80.38 | 71.21 | 0.36 |
| 0.6 | 477 | 206 | 1894 | 723 | 39.75 | 90.19 | 71.85 | 0.36 |
| 0.7 | 413 | 161 | 1939 | 787 | 34.42 | 92.33 | 71.27 | 0.34 |
| 0.8 | 390 | 146 | 1954 | 810 | 32.5 | 93.05 | 71.03 | 0.33 |
| 0.9 | 382 | 144 | 1956 | 818 | 31.83 | 93.14 | 70.85 | 0.33 |
| 1 | 379 | 139 | 1961 | 821 | 31.58 | 93.38 | 70.91 | 0.33 |
