# Supplementary material for: Improved Method for Linear B-Cell Epitope Prediction Using Antigen’s Primary Sequence
Source: PLoS One. 2013 May 7;8(5):e62216. doi: 10.1371/journal.pone.0062216 (PMC3646881; doi:10.1371/journal.pone.0062216)
Supplement: Table S7 — The performance of SVM/IBK models developed on Lbtope_Fixed dataset using AAP profile. These models were developed using 5-fold cross-validation on 90% data and tested on remaining 10% data. (DOC) [file pone.0062216.s010.doc]

**Table S7. The performance of SVM/IBK models developed on Lbtope_Fixed dataset using AAP profile. These models were developed using 5-fold cross-validation on 90% data and tested on remaining 10% data.**

| **SVM** | | | | | | | | |
| --- | --- | --- | --- | --- | --- | --- | --- | --- |
| **Thres** | **TP** | **FP** | **TN** | **FN** | **Sen** | **Spec** | **Accuracy** | **MCC** |
| -1 | 1197 | 1887 | 213 | 3 | 99.75 | 10.14 | 42.73 | 0.19 |
| -0.9 | 1172 | 1374 | 726 | 28 | 97.67 | 34.57 | 57.52 | 0.37 |
| -0.8 | 1156 | 1147 | 953 | 44 | 96.33 | 45.38 | 63.91 | 0.44 |
| -0.7 | 1143 | 988 | 1112 | 57 | 95.25 | 52.95 | 68.33 | 0.48 |
| -0.6 | 1126 | 880 | 1220 | 74 | 93.83 | 58.1 | 71.09 | 0.51 |
| -0.5 | 1116 | 763 | 1337 | 84 | 93 | 63.67 | 74.33 | 0.55 |
| -0.4 | 1085 | 670 | 1430 | 115 | 90.42 | 68.1 | 76.21 | 0.56 |
| -0.3 | 1041 | 569 | 1531 | 159 | 86.75 | 72.9 | 77.94 | 0.57 |
| -0.2 | 981 | 470 | 1630 | 219 | 81.75 | 77.62 | 79.12 | 0.58 |
| -0.1 | 867 | 359 | 1741 | 333 | 72.25 | 82.9 | 79.03 | 0.55 |
| 0 | 753 | 268 | 1832 | 447 | 62.75 | 87.24 | 78.33 | 0.52 |
| 0.1 | 664 | 221 | 1879 | 536 | 55.33 | 89.48 | 77.06 | 0.49 |
| 0.2 | 596 | 176 | 1924 | 604 | 49.67 | 91.62 | 76.36 | 0.47 |
| 0.3 | 541 | 148 | 1952 | 659 | 45.08 | 92.95 | 75.55 | 0.45 |
| 0.4 | 484 | 127 | 1973 | 716 | 40.33 | 93.95 | 74.45 | 0.42 |
| 0.5 | 433 | 100 | 2000 | 767 | 36.08 | 95.24 | 73.73 | 0.41 |
| 0.6 | 388 | 86 | 2014 | 812 | 32.33 | 95.9 | 72.79 | 0.39 |
| 0.7 | 338 | 69 | 2031 | 862 | 28.17 | 96.71 | 71.79 | 0.36 |
| 0.8 | 266 | 52 | 2048 | 934 | 22.17 | 97.52 | 70.12 | 0.32 |
| 0.9 | 192 | 35 | 2065 | 1008 | 16 | 98.33 | 68.39 | 0.27 |
| 1 | 65 | 11 | 2089 | 1135 | 5.42 | 99.48 | 65.27 | 0.16 |
| IBK | | | | | | | | |
| 0 | 1200 | 2100 | 0 | 0 | 100 | 0 | 36.36 | 0 |
| 0.1 | 986 | 472 | 1628 | 214 | 82.17 | 77.52 | 79.21 | 0.58 |
| 0.2 | 982 | 460 | 1640 | 218 | 81.83 | 78.1 | 79.45 | 0.58 |
| 0.3 | 970 | 431 | 1669 | 230 | 80.83 | 79.48 | 79.97 | 0.59 |
| 0.4 | 939 | 383 | 1717 | 261 | 78.25 | 81.76 | 80.48 | 0.59 |
| 0.5 | 844 | 282 | 1818 | 356 | 70.33 | 86.57 | 80.67 | 0.58 |
| 0.6 | 714 | 183 | 1917 | 486 | 59.5 | 91.29 | 79.73 | 0.55 |
| 0.7 | 660 | 149 | 1951 | 540 | 55 | 92.9 | 79.12 | 0.54 |
| 0.8 | 630 | 134 | 1966 | 570 | 52.5 | 93.62 | 78.67 | 0.53 |
| 0.9 | 614 | 127 | 1973 | 586 | 51.17 | 93.95 | 78.39 | 0.52 |
| 1 | 609 | 122 | 1978 | 591 | 50.75 | 94.19 | 78.39 | 0.52 |
