# Supplementary material for: Improved Method for Linear B-Cell Epitope Prediction Using Antigen’s Primary Sequence
Source: PLoS One. 2013 May 7;8(5):e62216. doi: 10.1371/journal.pone.0062216 (PMC3646881; doi:10.1371/journal.pone.0062216)
Supplement: Table S8 — The performance of SVM/IBK models developed on Lbtope_Fixed dataset using AAA profile. These models were developed using 5-fold cross-validation on 90% data and tested on remaining 10% data. (DOC) [file pone.0062216.s011.doc]

**Table S8. The performance of SVM/IBK models developed on Lbtope_Fixed dataset using AAA profile. These models were developed using 5-fold cross-validation on 90% data and tested on remaining 10% data.**

| **SVM** | | | | | | | | |
| --- | --- | --- | --- | --- | --- | --- | --- | --- |
| **Thres** | **TP** | **FP** | **TN** | **FN** | **Sen** | **Spec** | **Accuracy** | **MCC** |
| -1 | 1182 | 1981 | 119 | 18 | 98.5 | 5.67 | 39.42 | 0.1 |
| -0.9 | 1170 | 1926 | 174 | 30 | 97.5 | 8.29 | 40.73 | 0.12 |
| -0.8 | 1156 | 1853 | 247 | 44 | 96.33 | 11.76 | 42.52 | 0.14 |
| -0.7 | 1136 | 1778 | 322 | 64 | 94.67 | 15.33 | 44.18 | 0.15 |
| -0.6 | 1110 | 1704 | 396 | 90 | 92.5 | 18.86 | 45.64 | 0.15 |
| -0.5 | 1079 | 1607 | 493 | 121 | 89.92 | 23.48 | 47.64 | 0.17 |
| -0.4 | 1039 | 1499 | 601 | 161 | 86.58 | 28.62 | 49.7 | 0.17 |
| -0.3 | 991 | 1384 | 716 | 209 | 82.58 | 34.1 | 51.73 | 0.18 |
| -0.2 | 934 | 1253 | 847 | 266 | 77.83 | 40.33 | 53.97 | 0.18 |
| -0.1 | 864 | 1115 | 985 | 336 | 72 | 46.9 | 56.03 | 0.19 |
| 0 | 800 | 977 | 1123 | 400 | 66.67 | 53.48 | 58.27 | 0.19 |
| 0.1 | 738 | 871 | 1229 | 462 | 61.5 | 58.52 | 59.61 | 0.19 |
| 0.2 | 660 | 739 | 1361 | 540 | 55 | 64.81 | 61.24 | 0.19 |
| 0.3 | 594 | 638 | 1462 | 606 | 49.5 | 69.62 | 62.3 | 0.19 |
| 0.4 | 521 | 530 | 1570 | 679 | 43.42 | 74.76 | 63.36 | 0.19 |
| 0.5 | 453 | 423 | 1677 | 747 | 37.75 | 79.86 | 64.55 | 0.19 |
| 0.6 | 407 | 345 | 1755 | 793 | 33.92 | 83.57 | 65.52 | 0.2 |
| 0.7 | 353 | 284 | 1816 | 847 | 29.42 | 86.48 | 65.73 | 0.19 |
| 0.8 | 309 | 232 | 1868 | 891 | 25.75 | 88.95 | 65.97 | 0.19 |
| 0.9 | 266 | 194 | 1906 | 934 | 22.17 | 90.76 | 65.82 | 0.18 |
| 1 | 229 | 157 | 1943 | 971 | 19.08 | 92.52 | 65.82 | 0.17 |
