# Supplementary material for: Improved Method for Linear B-Cell Epitope Prediction Using Antigen’s Primary Sequence
Source: PLoS One. 2013 May 7;8(5):e62216. doi: 10.1371/journal.pone.0062216 (PMC3646881; doi:10.1371/journal.pone.0062216)
Supplement: Table S9 — The performance of SVM/IBK models developed on Lbtope_Fixed dataset using Dipeptide composition. These models were developed using 5-fold cross-validation on 90% data and tested on remaining 10% data. (DOC) [file pone.0062216.s012.doc]

**Table S9. The performance of SVM/IBK models developed on Lbtope_Fixed dataset using Dipeptide composition. These models were developed using 5-fold cross-validation on 90% data and tested on remaining 10% data.**

| **SVM** | | | | | | | | | |
| --- | --- | --- | --- | --- | --- | --- | --- | --- | --- |
| **Thres** | **TP** | **FP** | **TN** | **FN** | **Sen** | **Spec** | **Accuracy** | **MCC** |  |
| -1 | 1160 | 1231 | 869 | 40 | 96.67 | 41.38 | 61.48 | 0.41 |  |
| -0.9 | 1139 | 995 | 1105 | 61 | 94.92 | 52.62 | 68 | 0.48 |  |
| -0.8 | 1113 | 810 | 1290 | 87 | 92.75 | 61.43 | 72.82 | 0.53 |  |
| -0.7 | 1083 | 691 | 1409 | 117 | 90.25 | 67.1 | 75.52 | 0.55 |  |
| -0.6 | 1060 | 585 | 1515 | 140 | 88.33 | 72.14 | 78.03 | 0.58 |  |
| -0.5 | 1033 | 498 | 1602 | 167 | 86.08 | 76.29 | 79.85 | 0.6 |  |
| -0.4 | 1000 | 434 | 1666 | 200 | 83.33 | 79.33 | 80.79 | 0.61 |  |
| -0.3 | 966 | 385 | 1715 | 234 | 80.5 | 81.67 | 81.24 | 0.61 | ** |
| -0.2 | 921 | 327 | 1773 | 279 | 76.75 | 84.43 | 81.64 | 0.61 |  |
| -0.1 | 881 | 265 | 1835 | 319 | 73.42 | 87.38 | 82.3 | 0.61 |  |
| 0 | 839 | 236 | 1864 | 361 | 69.92 | 88.76 | 81.91 | 0.6 |  |
| 0.1 | 790 | 201 | 1899 | 410 | 65.83 | 90.43 | 81.48 | 0.59 |  |
| 0.2 | 731 | 175 | 1925 | 469 | 60.92 | 91.67 | 80.48 | 0.57 |  |
| 0.3 | 680 | 146 | 1954 | 520 | 56.67 | 93.05 | 79.82 | 0.55 |  |
| 0.4 | 613 | 118 | 1982 | 587 | 51.08 | 94.38 | 78.64 | 0.53 |  |
| 0.5 | 558 | 100 | 2000 | 642 | 46.5 | 95.24 | 77.52 | 0.5 |  |
| 0.6 | 495 | 83 | 2017 | 705 | 41.25 | 96.05 | 76.12 | 0.47 |  |
| 0.7 | 429 | 67 | 2033 | 771 | 35.75 | 96.81 | 74.61 | 0.44 |  |
| 0.8 | 373 | 55 | 2045 | 827 | 31.08 | 97.38 | 73.27 | 0.41 |  |
| 0.9 | 303 | 44 | 2056 | 897 | 25.25 | 97.9 | 71.48 | 0.36 |  |
| 1 | 224 | 25 | 2075 | 976 | 18.67 | 98.81 | 69.67 | 0.32 |  |
| IBK | | | | | | | | | |
| 0 | 1200 | 2100 | 0 | 0 | 100 | 0 | 36.36 | 0 |  |
| 0.1 | 1028 | 482 | 1618 | 172 | 85.67 | 77.05 | 80.18 | 0.61 |  |
| 0.2 | 1007 | 465 | 1635 | 193 | 83.92 | 77.86 | 80.06 | 0.6 |  |
| 0.3 | 964 | 385 | 1715 | 236 | 80.33 | 81.67 | 81.18 | 0.61 |  |
| 0.4 | 917 | 336 | 1764 | 283 | 76.42 | 84 | 81.24 | 0.6 |  |
| 0.5 | 874 | 280 | 1820 | 326 | 72.83 | 86.67 | 81.64 | 0.6 |  |
| 0.6 | 707 | 150 | 1950 | 493 | 58.92 | 92.86 | 80.52 | 0.57 |  |
| 0.7 | 639 | 112 | 1988 | 561 | 53.25 | 94.67 | 79.61 | 0.55 |  |
| 0.8 | 589 | 95 | 2005 | 611 | 49.08 | 95.48 | 78.61 | 0.53 |  |
| 0.9 | 567 | 87 | 2013 | 633 | 47.25 | 95.86 | 78.18 | 0.52 |  |
| 1 | 561 | 84 | 2016 | 639 | 46.75 | 96 | 78.09 | 0.52 |  |
