# Supplementary material for: Improved Method for Linear B-Cell Epitope Prediction Using Antigen’s Primary Sequence
Source: PLoS One. 2013 May 7;8(5):e62216. doi: 10.1371/journal.pone.0062216 (PMC3646881; doi:10.1371/journal.pone.0062216)
Supplement: Table S10 — The performance of SVM/IBK models developed on Lbtope_Variable dataset using Amino acid composition. These models were developed using 5-fold cross-validation on 90% data and tested on remaining 10% data. (DOC) [file pone.0062216.s013.doc]

**Table S10. The performance of SVM /IBK models developed on Lbtope_Variable dataset using Amino acid composition. These models were developed using 5-fold cross-validation on 90% data and tested on remaining 10% data.**

| **SVM** | | | | | | | | |
| --- | --- | --- | --- | --- | --- | --- | --- | --- |
| **Thres** | **TP** | **FP** | **TN** | **FN** | **Sen** | **Spec** | **Accuracy** | **MCC** |
| -1 | 1478 | 2267 | 66 | 9 | 99.39 | 2.83 | 40.42 | 0.08 |
| -0.9 | 1465 | 2138 | 195 | 22 | 98.52 | 8.36 | 43.46 | 0.14 |
| -0.8 | 1460 | 2107 | 226 | 27 | 98.18 | 9.69 | 44.14 | 0.15 |
| -0.7 | 1436 | 2010 | 323 | 51 | 96.57 | 13.84 | 46.05 | 0.17 |
| -0.6 | 1395 | 1816 | 517 | 92 | 93.81 | 22.16 | 50.05 | 0.21 |
| -0.5 | 1307 | 1401 | 932 | 180 | 87.9 | 39.95 | 58.61 | 0.3 |
| -0.4 | 1012 | 786 | 1547 | 475 | 68.06 | 66.31 | 66.99 | 0.34 |
| -0.3 | 804 | 515 | 1818 | 683 | 54.07 | 77.93 | 68.64 | 0.33 |
| -0.2 | 665 | 361 | 1972 | 822 | 44.72 | 84.53 | 69.03 | 0.32 |
| -0.1 | 551 | 249 | 2084 | 936 | 37.05 | 89.33 | 68.98 | 0.32 |
| 0 | 452 | 167 | 2166 | 1035 | 30.4 | 92.84 | 68.53 | 0.31 |
| 0.1 | 393 | 132 | 2201 | 1094 | 26.43 | 94.34 | 67.91 | 0.29 |
| 0.2 | 333 | 96 | 2237 | 1154 | 22.39 | 95.89 | 67.28 | 0.28 |
| 0.3 | 281 | 72 | 2261 | 1206 | 18.9 | 96.91 | 66.54 | 0.27 |
| 0.4 | 241 | 54 | 2279 | 1246 | 16.21 | 97.69 | 65.97 | 0.25 |
| 0.5 | 208 | 37 | 2296 | 1279 | 13.99 | 98.41 | 65.55 | 0.25 |
| 0.6 | 175 | 23 | 2310 | 1312 | 11.77 | 99.01 | 65.05 | 0.24 |
| 0.7 | 141 | 15 | 2318 | 1346 | 9.48 | 99.36 | 64.37 | 0.22 |
| 0.8 | 107 | 11 | 2322 | 1380 | 7.2 | 99.53 | 63.59 | 0.19 |
| 0.9 | 95 | 9 | 2324 | 1392 | 6.39 | 99.61 | 63.32 | 0.18 |
| 1 | 69 | 3 | 2330 | 1418 | 4.64 | 99.87 | 62.8 | 0.16 |
| IBK | | | | | | | | |
| 0 | 1487 | 2333 | 0 | 0 | 100 | 0 | 38.93 | 0 |
| 0.1 | 1188 | 967 | 1366 | 299 | 79.89 | 58.55 | 66.86 | 0.38 |
| 0.2 | 1168 | 899 | 1434 | 319 | 78.55 | 61.47 | 68.12 | 0.39 |
| 0.3 | 1123 | 774 | 1559 | 364 | 75.52 | 66.82 | 70.21 | 0.41 |
| 0.4 | 1047 | 634 | 1699 | 440 | 70.41 | 72.82 | 71.88 | 0.42 |
| 0.5 | 907 | 479 | 1854 | 580 | 61 | 79.47 | 72.28 | 0.41 |
| 0.6 | 664 | 248 | 2085 | 823 | 44.65 | 89.37 | 71.96 | 0.39 |
| 0.7 | 579 | 190 | 2143 | 908 | 38.94 | 91.86 | 71.26 | 0.37 |
| 0.8 | 539 | 172 | 2161 | 948 | 36.25 | 92.63 | 70.68 | 0.36 |
| 0.9 | 520 | 167 | 2166 | 967 | 34.97 | 92.84 | 70.31 | 0.35 |
| 1 | 511 | 166 | 2167 | 976 | 34.36 | 92.88 | 70.1 | 0.35 |
