# Supplementary material for: Improved Method for Linear B-Cell Epitope Prediction Using Antigen’s Primary Sequence
Source: PLoS One. 2013 May 7;8(5):e62216. doi: 10.1371/journal.pone.0062216 (PMC3646881; doi:10.1371/journal.pone.0062216)
Supplement: Table S11 — The performance of SVM/IBK models developed on Lbtope_Variable dataset using Composition Transition. These models were developed using 5-fold cross-validation on 90% data and tested on remaining 10% data. (DOC) [file pone.0062216.s014.doc]

**Table S11. The performance of SVM/IBK models developed on Lbtope_Variable dataset using Composition Transition. These models were developed using 5-fold cross-validation on 90% data and tested on remaining 10% data.**

| **SVM** | | | | | | | | |
| --- | --- | --- | --- | --- | --- | --- | --- | --- |
| **Thres** | **TP** | **FP** | **TN** | **FN** | **Sen** | **Spec** | **Accuracy** | **MCC** |
| -1 | 1475 | 2198 | 135 | 12 | 99.19 | 5.79 | 42.15 | 0.13 |
| -0.9 | 1465 | 2110 | 223 | 22 | 98.52 | 9.56 | 44.19 | 0.16 |
| -0.8 | 1448 | 2039 | 294 | 39 | 97.38 | 12.6 | 45.6 | 0.17 |
| -0.7 | 1438 | 1940 | 393 | 49 | 96.7 | 16.85 | 47.93 | 0.21 |
| -0.6 | 1413 | 1810 | 523 | 74 | 95.02 | 22.42 | 50.68 | 0.23 |
| -0.5 | 1386 | 1670 | 663 | 101 | 93.21 | 28.42 | 53.64 | 0.26 |
| -0.4 | 1352 | 1526 | 807 | 135 | 90.92 | 34.59 | 56.52 | 0.29 |
| -0.3 | 1295 | 1370 | 963 | 192 | 87.09 | 41.28 | 59.11 | 0.3 |
| -0.2 | 1213 | 1185 | 1148 | 274 | 81.57 | 49.21 | 61.81 | 0.31 |
| -0.1 | 1007 | 830 | 1503 | 480 | 67.72 | 64.42 | 65.71 | 0.31 |
| 0 | 300 | 131 | 2202 | 1187 | 20.17 | 94.38 | 65.5 | 0.22 |
| 0.1 | 223 | 84 | 2249 | 1264 | 15 | 96.4 | 64.71 | 0.2 |
| 0.2 | 180 | 67 | 2266 | 1307 | 12.1 | 97.13 | 64.03 | 0.18 |
| 0.3 | 145 | 53 | 2280 | 1342 | 9.75 | 97.73 | 63.48 | 0.16 |
| 0.4 | 118 | 41 | 2292 | 1369 | 7.94 | 98.24 | 63.09 | 0.15 |
| 0.5 | 94 | 36 | 2297 | 1393 | 6.32 | 98.46 | 62.59 | 0.13 |
| 0.6 | 77 | 28 | 2305 | 1410 | 5.18 | 98.8 | 62.36 | 0.12 |
| 0.7 | 70 | 27 | 2306 | 1417 | 4.71 | 98.84 | 62.2 | 0.11 |
| 0.8 | 64 | 23 | 2310 | 1423 | 4.3 | 99.01 | 62.15 | 0.11 |
| 0.9 | 58 | 18 | 2315 | 1429 | 3.9 | 99.23 | 62.12 | 0.11 |
| 1 | 25 | 9 | 2324 | 1462 | 1.68 | 99.61 | 61.49 | 0.07 |
| IBK | | | | | | | | |
| 0 | 1487 | 2333 | 0 | 0 | 100 | 0 | 38.93 | 0 |
| 0.1 | 1115 | 1154 | 1179 | 372 | 74.98 | 50.54 | 60.05 | 0.25 |
| 0.2 | 1112 | 1140 | 1193 | 375 | 74.78 | 51.14 | 60.34 | 0.26 |
| 0.3 | 1088 | 1064 | 1269 | 399 | 73.17 | 54.39 | 61.7 | 0.27 |
| 0.4 | 987 | 907 | 1426 | 500 | 66.38 | 61.12 | 63.17 | 0.27 |
| 0.5 | 823 | 654 | 1679 | 664 | 55.35 | 71.97 | 65.5 | 0.27 |
| 0.6 | 581 | 325 | 2008 | 906 | 39.07 | 86.07 | 67.77 | 0.29 |
| 0.7 | 495 | 224 | 2109 | 992 | 33.29 | 90.4 | 68.17 | 0.3 |
| 0.8 | 473 | 206 | 2127 | 1014 | 31.81 | 91.17 | 68.06 | 0.29 |
| 0.9 | 464 | 203 | 2130 | 1023 | 31.2 | 91.3 | 67.91 | 0.29 |
| 1 | 460 | 200 | 2133 | 1027 | 30.93 | 91.43 | 67.88 | 0.29 |
