# Supplementary material for: Improved Method for Linear B-Cell Epitope Prediction Using Antigen’s Primary Sequence
Source: PLoS One. 2013 May 7;8(5):e62216. doi: 10.1371/journal.pone.0062216 (PMC3646881; doi:10.1371/journal.pone.0062216)
Supplement: Table S12 — The performance of SVM/IBK models developed on Lbtope_Variable dataset using AAP profile. These models were developed using 5-fold cross-validation on 90% data and tested on remaining 10% data. (DOC) [file pone.0062216.s015.doc]

**Table S12. The performance of SVM/IBK models developed on Lbtope_Variable dataset using AAP profile. These models were developed using 5-fold cross-validation on 90% data and tested on remaining 10% data.**

| **SVM** | | | | | | | | |
| --- | --- | --- | --- | --- | --- | --- | --- | --- |
| **Thres** | **TP** | **FP** | **TN** | **FN** | **Sen** | **Spec** | **Accuracy** | **MCC** |
| -1 | 1455 | 2057 | 276 | 32 | 97.85 | 11.83 | 45.31 | 0.17 |
| -0.9 | 1441 | 1911 | 422 | 46 | 96.91 | 18.09 | 48.77 | 0.22 |
| -0.8 | 1422 | 1777 | 556 | 65 | 95.63 | 23.83 | 51.78 | 0.26 |
| -0.7 | 1400 | 1659 | 674 | 87 | 94.15 | 28.89 | 54.29 | 0.28 |
| -0.6 | 1380 | 1534 | 799 | 107 | 92.8 | 34.25 | 57.04 | 0.31 |
| -0.5 | 1354 | 1413 | 920 | 133 | 91.06 | 39.43 | 59.53 | 0.33 |
| -0.4 | 1328 | 1289 | 1044 | 159 | 89.31 | 44.75 | 62.09 | 0.36 |
| -0.3 | 1286 | 1154 | 1179 | 201 | 86.48 | 50.54 | 64.53 | 0.38 |
| -0.2 | 1239 | 1049 | 1284 | 248 | 83.32 | 55.04 | 66.05 | 0.38 |
| -0.1 | 1179 | 942 | 1391 | 308 | 79.29 | 59.62 | 67.28 | 0.38 |
| 0 | 1131 | 843 | 1490 | 356 | 76.06 | 63.87 | 68.61 | 0.39 |
| 0.1 | 1060 | 729 | 1604 | 427 | 71.28 | 68.75 | 69.74 | 0.39 |
| 0.2 | 1004 | 629 | 1704 | 483 | 67.52 | 73.04 | 70.89 | 0.4 |
| 0.3 | 925 | 545 | 1788 | 562 | 62.21 | 76.64 | 71.02 | 0.39 |
| 0.4 | 854 | 466 | 1867 | 633 | 57.43 | 80.03 | 71.23 | 0.38 |
| 0.5 | 771 | 383 | 1950 | 716 | 51.85 | 83.58 | 71.23 | 0.38 |
| 0.6 | 692 | 311 | 2022 | 795 | 46.54 | 86.67 | 71.05 | 0.37 |
| 0.7 | 611 | 247 | 2086 | 876 | 41.09 | 89.41 | 70.6 | 0.36 |
| 0.8 | 533 | 184 | 2149 | 954 | 35.84 | 92.11 | 70.21 | 0.35 |
| 0.9 | 446 | 128 | 2205 | 1041 | 29.99 | 94.51 | 69.4 | 0.33 |
| 1 | 347 | 87 | 2246 | 1140 | 23.34 | 96.27 | 67.88 | 0.3 |
| IBK | | | | | | | | |
| 0 | 1487 | 2333 | 0 | 0 | 100 | 0 | 38.93 | 0 |
| 0.1 | 1221 | 779 | 1554 | 266 | 82.11 | 66.61 | 72.64 | 0.48 |
| 0.2 | 1208 | 738 | 1595 | 279 | 81.24 | 68.37 | 73.38 | 0.48 |
| 0.3 | 1155 | 544 | 1789 | 332 | 77.67 | 76.68 | 77.07 | 0.53 |
| 0.4 | 871 | 290 | 2043 | 616 | 58.57 | 87.57 | 76.28 | 0.49 |
| 0.5 | 813 | 238 | 2095 | 674 | 54.67 | 89.8 | 76.13 | 0.49 |
| 0.6 | 770 | 200 | 2133 | 717 | 51.78 | 91.43 | 75.99 | 0.48 |
| 0.7 | 624 | 103 | 2230 | 863 | 41.96 | 95.59 | 74.71 | 0.47 |
| 0.8 | 510 | 70 | 2263 | 977 | 34.3 | 97 | 72.59 | 0.43 |
| 0.9 | 452 | 54 | 2279 | 1035 | 30.4 | 97.69 | 71.49 | 0.4 |
| 1 | 440 | 54 | 2279 | 1047 | 29.59 | 97.69 | 71.18 | 0.4 |
