# Supplementary material for: Improved Method for Linear B-Cell Epitope Prediction Using Antigen’s Primary Sequence
Source: PLoS One. 2013 May 7;8(5):e62216. doi: 10.1371/journal.pone.0062216 (PMC3646881; doi:10.1371/journal.pone.0062216)
Supplement: Table S13 — The performance of SVM/IBK models developed on Lbtope_Variable dataset using Dipeptide composition. These models were developed using 5-fold cross-validation on 90% data and tested on remaining 10% data. (DOC) [file pone.0062216.s016.doc]

**Table S13. The performance of SVM/IBK models developed on Lbtope_Variable dataset using Dipeptide composition. These models were developed using 5-fold cross-validation on 90% data and tested on remaining 10% data.**

| **SVM** | | | | | | | | | |
| --- | --- | --- | --- | --- | --- | --- | --- | --- | --- |
| **Thres** | **TP** | **FP** | **TN** | **FN** | **Sen** | **Spec** | **Accuracy** | **MCC** |  |
| -1 | 1443 | 1776 | 557 | 44 | 97.04 | 23.87 | 52.36 | 0.28 |  |
| -0.9 | 1414 | 1566 | 767 | 73 | 95.09 | 32.88 | 57.09 | 0.33 |  |
| -0.8 | 1394 | 1357 | 976 | 93 | 93.75 | 41.83 | 62.04 | 0.39 |  |
| -0.7 | 1353 | 1177 | 1156 | 134 | 90.99 | 49.55 | 65.68 | 0.42 |  |
| -0.6 | 1325 | 1011 | 1322 | 162 | 89.11 | 56.67 | 69.29 | 0.46 |  |
| -0.5 | 1287 | 867 | 1466 | 200 | 86.55 | 62.84 | 72.07 | 0.49 |  |
| -0.4 | 1241 | 746 | 1587 | 246 | 83.46 | 68.02 | 74.03 | 0.5 |  |
| -0.3 | 1188 | 646 | 1687 | 299 | 79.89 | 72.31 | 75.26 | 0.51 |  |
| -0.2 | 1118 | 552 | 1781 | 369 | 75.18 | 76.34 | 75.89 | 0.51 | ** |
| -0.1 | 1052 | 464 | 1869 | 435 | 70.75 | 80.11 | 76.47 | 0.51 |  |
| 0 | 994 | 388 | 1945 | 493 | 66.85 | 83.37 | 76.94 | 0.51 |  |
| 0.1 | 909 | 323 | 2010 | 578 | 61.13 | 86.16 | 76.41 | 0.49 |  |
| 0.2 | 829 | 269 | 2064 | 658 | 55.75 | 88.47 | 75.73 | 0.48 |  |
| 0.3 | 759 | 213 | 2120 | 728 | 51.04 | 90.87 | 75.37 | 0.47 |  |
| 0.4 | 690 | 167 | 2166 | 797 | 46.4 | 92.84 | 74.76 | 0.46 |  |
| 0.5 | 620 | 129 | 2204 | 867 | 41.69 | 94.47 | 73.93 | 0.44 |  |
| 0.6 | 541 | 96 | 2237 | 946 | 36.38 | 95.89 | 72.72 | 0.42 |  |
| 0.7 | 466 | 74 | 2259 | 1021 | 31.34 | 96.83 | 71.34 | 0.39 |  |
| 0.8 | 391 | 53 | 2280 | 1096 | 26.29 | 97.73 | 69.92 | 0.37 |  |
| 0.9 | 303 | 30 | 2303 | 1184 | 20.38 | 98.71 | 68.22 | 0.33 |  |
| 1 | 225 | 18 | 2315 | 1262 | 15.13 | 99.23 | 66.49 | 0.29 |  |
| IBK | | | | | | | | | |
| 0 | 13389 | 20988 | 0 | 0 | 100 | 0 | 38.95 | 0 |  |
| 0.1 | 11161 | 5857 | 15131 | 2228 | 83.36 | 72.09 | 76.48 | 0.54 |  |
| 0.2 | 11057 | 5710 | 15278 | 2332 | 82.58 | 72.79 | 76.61 | 0.54 |  |
| 0.3 | 10772 | 5109 | 15879 | 2617 | 80.45 | 75.66 | 77.53 | 0.55 |  |
| 0.4 | 10253 | 4333 | 16655 | 3136 | 76.58 | 79.35 | 78.27 | 0.55 |  |
| 0.5 | 9385 | 3334 | 17654 | 4004 | 70.09 | 84.11 | 78.65 | 0.55 |  |
| 0.6 | 7448 | 1740 | 19248 | 5941 | 55.63 | 91.71 | 77.66 | 0.52 |  |
| 0.7 | 6731 | 1350 | 19638 | 6658 | 50.27 | 93.57 | 76.71 | 0.5 |  |
| 0.8 | 6387 | 1197 | 19791 | 7002 | 47.7 | 94.3 | 76.15 | 0.49 |  |
| 0.9 | 6252 | 1159 | 19829 | 7137 | 46.7 | 94.48 | 75.87 | 0.49 |  |
| 1 | 6214 | 1157 | 19831 | 7175 | 46.41 | 94.49 | 75.76 | 0.49 |  |
