# Supplementary material for: Improved Method for Linear B-Cell Epitope Prediction Using Antigen’s Primary Sequence
Source: PLoS One. 2013 May 7;8(5):e62216. doi: 10.1371/journal.pone.0062216 (PMC3646881; doi:10.1371/journal.pone.0062216)
Supplement: Table S14 — The performance of SVM/IBK models developed on Lbtope_Confirm (epitope tested by at least two studies) dataset using Amino acid composition. These models were developed using 5-fold cross-validation on 90% data and tested on remaining 10% data. (DOC) [file pone.0062216.s017.doc]

**Table S14. The performance of SVM/IBK models developed on Lbtope_Confirm (epitope tested by at least two studies) dataset using Amino acid composition. These models were developed using 5-fold cross-validation on 90% data and tested on remaining 10% data.**

| **SVM** | | | | | | | | |
| --- | --- | --- | --- | --- | --- | --- | --- | --- |
| **Thres** | **TP** | **FP** | **TN** | **FN** | **Sen** | **Spec** | **Accuracy** | **MCC** |
| -1 | 100 | 119 | 60 | 4 | 96.15 | 33.52 | 56.54 | 0.34 |
| -0.9 | 100 | 111 | 68 | 4 | 96.15 | 37.99 | 59.36 | 0.38 |
| -0.8 | 99 | 100 | 79 | 5 | 95.19 | 44.13 | 62.9 | 0.42 |
| -0.7 | 97 | 97 | 82 | 7 | 93.27 | 45.81 | 63.25 | 0.41 |
| -0.6 | 97 | 87 | 92 | 7 | 93.27 | 51.4 | 66.78 | 0.45 |
| -0.5 | 95 | 78 | 101 | 9 | 91.35 | 56.42 | 69.26 | 0.47 |
| -0.4 | 94 | 73 | 106 | 10 | 90.38 | 59.22 | 70.67 | 0.49 |
| -0.3 | 93 | 65 | 114 | 11 | 89.42 | 63.69 | 73.14 | 0.52 |
| -0.2 | 93 | 58 | 121 | 11 | 89.42 | 67.6 | 75.62 | 0.55 |
| -0.1 | 90 | 55 | 124 | 14 | 86.54 | 69.27 | 75.62 | 0.54 |
| 0 | 85 | 48 | 131 | 19 | 81.73 | 73.18 | 76.33 | 0.53 |
| 0.1 | 77 | 41 | 138 | 27 | 74.04 | 77.09 | 75.97 | 0.5 |
| 0.2 | 71 | 34 | 145 | 33 | 68.27 | 81.01 | 76.33 | 0.49 |
| 0.3 | 67 | 30 | 149 | 37 | 64.42 | 83.24 | 76.33 | 0.48 |
| 0.4 | 62 | 25 | 154 | 42 | 59.62 | 86.03 | 76.33 | 0.48 |
| 0.5 | 58 | 23 | 156 | 46 | 55.77 | 87.15 | 75.62 | 0.46 |
| 0.6 | 54 | 18 | 161 | 50 | 51.92 | 89.94 | 75.97 | 0.46 |
| 0.7 | 48 | 13 | 166 | 56 | 46.15 | 92.74 | 75.62 | 0.46 |
| 0.8 | 43 | 9 | 170 | 61 | 41.35 | 94.97 | 75.27 | 0.45 |
| 0.9 | 36 | 8 | 171 | 68 | 34.62 | 95.53 | 73.14 | 0.4 |
| 1 | 34 | 5 | 174 | 70 | 32.69 | 97.21 | 73.5 | 0.42 |
| IBK | | | | | | | | |
| 0 | 104 | 179 | 0 | 0 | 100 | 0 | 36.75 | 0 |
| 0.1 | 87 | 48 | 131 | 17 | 83.65 | 73.18 | 77.03 | 0.55 |
| 0.2 | 86 | 46 | 133 | 18 | 82.69 | 74.3 | 77.39 | 0.55 |
| 0.3 | 84 | 41 | 138 | 20 | 80.77 | 77.09 | 78.45 | 0.56 |
| 0.4 | 81 | 33 | 146 | 23 | 77.88 | 81.56 | 80.21 | 0.58 |
| 0.5 | 72 | 19 | 160 | 32 | 69.23 | 89.39 | 81.98 | 0.61 |
| 0.6 | 57 | 6 | 173 | 47 | 54.81 | 96.65 | 81.27 | 0.6 |
| 0.7 | 51 | 6 | 173 | 53 | 49.04 | 96.65 | 79.15 | 0.55 |
| 0.8 | 49 | 5 | 174 | 55 | 47.12 | 97.21 | 78.8 | 0.54 |
| 0.9 | 49 | 5 | 174 | 55 | 47.12 | 97.21 | 78.8 | 0.54 |
| 1 | 1 | 0 | 179 | 103 | 0.96 | 100 | 63.6 | 0.08 |
