# Supplementary material for: Improved Method for Linear B-Cell Epitope Prediction Using Antigen’s Primary Sequence
Source: PLoS One. 2013 May 7;8(5):e62216. doi: 10.1371/journal.pone.0062216 (PMC3646881; doi:10.1371/journal.pone.0062216)
Supplement: Table S15 — The performance of SVM/IBK models developed on Lbtope_Confirm (epitope tested by at least two studies) dataset using Composition Transition. These models were developed using 5-fold cross-validation on 90% data and tested on remaining 10% data. (DOC) [file pone.0062216.s018.doc]

**Table S15. The performance of SVM/IBK models developed on Lbtope_Confirm (epitope tested by at least two studies) dataset using Composition Transition. These models were developed using 5-fold cross-validation on 90% data and tested on remaining 10% data.**

| **SVM** | | | | | | | | |
| --- | --- | --- | --- | --- | --- | --- | --- | --- |
| **Thres** | **TP** | **FP** | **TN** | **FN** | **Sen** | **Spec** | **Accuracy** | **MCC** |
| -1 | 100 | 114 | 65 | 4 | 96.15 | 36.31 | 58.3 | 0.36 |
| -0.9 | 100 | 102 | 77 | 4 | 96.15 | 43.02 | 62.54 | 0.42 |
| -0.8 | 96 | 94 | 85 | 8 | 92.31 | 47.49 | 63.96 | 0.41 |
| -0.7 | 96 | 83 | 96 | 8 | 92.31 | 53.63 | 67.84 | 0.46 |
| -0.6 | 94 | 76 | 103 | 10 | 90.38 | 57.54 | 69.61 | 0.47 |
| -0.5 | 92 | 71 | 108 | 12 | 88.46 | 60.34 | 70.67 | 0.48 |
| -0.4 | 91 | 65 | 114 | 13 | 87.5 | 63.69 | 72.44 | 0.5 |
| -0.3 | 87 | 59 | 120 | 17 | 83.65 | 67.04 | 73.14 | 0.49 |
| -0.2 | 85 | 53 | 126 | 19 | 81.73 | 70.39 | 74.56 | 0.50 |
| -0.1 | 80 | 45 | 134 | 24 | 76.92 | 74.86 | 75.62 | 0.50 |
| 0 | 74 | 39 | 140 | 30 | 71.15 | 78.21 | 75.62 | 0.49 |
| 0.1 | 65 | 34 | 145 | 39 | 62.5 | 81.01 | 74.2 | 0.44 |
| 0.2 | 61 | 30 | 149 | 43 | 58.65 | 83.24 | 74.2 | 0.43 |
| 0.3 | 58 | 23 | 156 | 46 | 55.77 | 87.15 | 75.62 | 0.46 |
| 0.4 | 50 | 19 | 160 | 54 | 48.08 | 89.39 | 74.2 | 0.42 |
| 0.5 | 45 | 13 | 166 | 59 | 43.27 | 92.74 | 74.56 | 0.43 |
| 0.6 | 40 | 11 | 168 | 64 | 38.46 | 93.85 | 73.5 | 0.41 |
| 0.7 | 33 | 9 | 170 | 71 | 31.73 | 94.97 | 71.73 | 0.36 |
| 0.8 | 25 | 9 | 170 | 79 | 24.04 | 94.97 | 68.9 | 0.28 |
| 0.9 | 20 | 7 | 172 | 84 | 19.23 | 96.09 | 67.84 | 0.25 |
| 1 | 12 | 5 | 174 | 92 | 11.54 | 97.21 | 65.72 | 0.18 |
| IBK | | | | | | | | |
| 0 | 938 | 1616 | 0 | 0 | 100 | 0 | 36.73 | 0 |
| 0.1 | 775 | 397 | 1219 | 163 | 82.62 | 75.43 | 78.07 | 0.56 |
| 0.2 | 769 | 374 | 1242 | 169 | 81.98 | 76.86 | 78.74 | 0.57 |
| 0.3 | 749 | 341 | 1275 | 189 | 79.85 | 78.9 | 79.25 | 0.57 |
| 0.4 | 725 | 284 | 1332 | 213 | 77.29 | 82.43 | 80.54 | 0.59 |
| 0.5 | 610 | 203 | 1413 | 328 | 65.03 | 87.44 | 79.21 | 0.54 |
| 0.6 | 451 | 96 | 1520 | 487 | 48.08 | 94.06 | 77.17 | 0.5 |
| 0.7 | 410 | 84 | 1532 | 528 | 43.71 | 94.8 | 76.04 | 0.47 |
| 0.8 | 391 | 78 | 1538 | 547 | 41.68 | 95.17 | 75.53 | 0.46 |
| 0.9 | 383 | 77 | 1539 | 555 | 40.83 | 95.24 | 75.25 | 0.45 |
| 1 | 19 | 1 | 1615 | 919 | 2.03 | 99.94 | 63.98 | 0.11 |
