# Supplementary material for: Improved Method for Linear B-Cell Epitope Prediction Using Antigen’s Primary Sequence
Source: PLoS One. 2013 May 7;8(5):e62216. doi: 10.1371/journal.pone.0062216 (PMC3646881; doi:10.1371/journal.pone.0062216)
Supplement: Table S16 — The performance of SVM/IBK models developed on Lbtope_Confirm (epitope tested by at least two studies) dataset using AAP profile. These models were developed using 5-fold cross-validation on 90% data and tested on remaining 10% data. (DOC) [file pone.0062216.s019.doc]

**Table S16. The performance of SVM/IBK models developed on Lbtope_Confirm (epitope tested by at least two studies) dataset using AAP profile. These models were developed using 5-fold cross-validation on 90% data and tested on remaining 10% data.**

| **SVM** | | | | | | | | |
| --- | --- | --- | --- | --- | --- | --- | --- | --- |
| **Thres** | **TP** | **FP** | **TN** | **FN** | **Sen** | **Spec** | **Accuracy** | **MCC** |
| -1 | 98 | 108 | 71 | 6 | 94.23 | 39.66 | 59.72 | 0.37 |
| -0.9 | 98 | 98 | 81 | 6 | 94.23 | 45.25 | 63.25 | 0.41 |
| -0.8 | 98 | 88 | 91 | 6 | 94.23 | 50.84 | 66.78 | 0.46 |
| -0.7 | 97 | 82 | 97 | 7 | 93.27 | 54.19 | 68.55 | 0.47 |
| -0.6 | 95 | 77 | 102 | 9 | 91.35 | 56.98 | 69.61 | 0.48 |
| -0.5 | 95 | 75 | 104 | 9 | 91.35 | 58.1 | 70.32 | 0.49 |
| -0.4 | 93 | 68 | 111 | 11 | 89.42 | 62.01 | 72.08 | 0.5 |
| -0.3 | 92 | 64 | 115 | 12 | 88.46 | 64.25 | 73.14 | 0.51 |
| -0.2 | 89 | 58 | 121 | 15 | 85.58 | 67.6 | 74.2 | 0.51 |
| -0.1 | 88 | 52 | 127 | 16 | 84.62 | 70.95 | 75.97 | 0.54 |
| 0 | 85 | 47 | 132 | 19 | 81.73 | 73.74 | 76.68 | 0.54 |
| 0.1 | 80 | 43 | 136 | 24 | 76.92 | 75.98 | 76.33 | 0.51 |
| 0.2 | 76 | 35 | 144 | 28 | 73.08 | 80.45 | 77.74 | 0.53 |
| 0.3 | 76 | 30 | 149 | 28 | 73.08 | 83.24 | 79.51 | 0.56 |
| 0.4 | 74 | 28 | 151 | 30 | 71.15 | 84.36 | 79.51 | 0.56 |
| 0.5 | 68 | 25 | 154 | 36 | 65.38 | 86.03 | 78.45 | 0.53 |
| 0.6 | 61 | 22 | 157 | 43 | 58.65 | 87.71 | 77.03 | 0.49 |
| 0.7 | 52 | 18 | 161 | 52 | 50 | 89.94 | 75.27 | 0.45 |
| 0.8 | 45 | 15 | 164 | 59 | 43.27 | 91.62 | 73.85 | 0.41 |
| 0.9 | 38 | 10 | 169 | 66 | 36.54 | 94.41 | 73.14 | 0.4 |
| 1 | 26 | 8 | 171 | 78 | 25 | 95.53 | 69.61 | 0.3 |
| IBK | | | | | | | | |
| 0 | 104 | 179 | 0 | 0 | 100 | 0 | 36.75 | 0 |
| 0.1 | 92 | 37 | 142 | 12 | 88.46 | 79.33 | 82.69 | 0.66 |
| 0.2 | 92 | 37 | 142 | 12 | 88.46 | 79.33 | 82.69 | 0.66 |
| 0.3 | 91 | 35 | 144 | 13 | 87.5 | 80.45 | 83.04 | 0.66 |
| 0.4 | 91 | 33 | 146 | 13 | 87.5 | 81.56 | 83.75 | 0.67 |
| 0.5 | 83 | 26 | 153 | 21 | 79.81 | 85.47 | 83.39 | 0.65 |
| 0.6 | 73 | 22 | 157 | 31 | 70.19 | 87.71 | 81.27 | 0.59 |
| 0.7 | 70 | 22 | 157 | 34 | 67.31 | 87.71 | 80.21 | 0.57 |
| 0.8 | 70 | 22 | 157 | 34 | 67.31 | 87.71 | 80.21 | 0.57 |
| 0.9 | 68 | 22 | 157 | 36 | 65.38 | 87.71 | 79.51 | 0.55 |
| 1 | 68 | 22 | 157 | 36 | 65.38 | 87.71 | 79.51 | 0.55 |
