# Supplementary material for: Improved Method for Linear B-Cell Epitope Prediction Using Antigen’s Primary Sequence
Source: PLoS One. 2013 May 7;8(5):e62216. doi: 10.1371/journal.pone.0062216 (PMC3646881; doi:10.1371/journal.pone.0062216)
Supplement: Table S17 — The performance of SVM/IBK models developed on Lbtope_Confirm (epitope tested by at least two studies) dataset using Dipeptide composition. These models were developed using 5-fold cross-validation on 90% data and tested on remaining 10% data. (DOC) [file pone.0062216.s020.doc]

**Table S17. The performance of SVM/IBK models developed on Lbtope_Confirm (epitope tested by at least two studies) dataset using Dipeptide composition. These models were developed using 5-fold cross-validation on 90% data and tested on remaining 10% data.**

| **SVM** | | | | | | | | | |
| --- | --- | --- | --- | --- | --- | --- | --- | --- | --- |
| **Thres** | **TP** | **FP** | **TN** | **FN** | **Sen** | **Spec** | **Accuracy** | **MCC** |  |
| -1 | 104 | 144 | 35 | 0 | 100 | 19.55 | 49.12 | 0.29 |  |
| -0.9 | 103 | 112 | 67 | 1 | 99.04 | 37.43 | 60.07 | 0.41 |  |
| -0.8 | 103 | 89 | 90 | 1 | 99.04 | 50.28 | 68.2 | 0.51 |  |
| -0.7 | 102 | 70 | 109 | 2 | 98.08 | 60.89 | 74.56 | 0.58 |  |
| -0.6 | 96 | 62 | 117 | 8 | 92.31 | 65.36 | 75.27 | 0.56 |  |
| -0.5 | 94 | 53 | 126 | 10 | 90.38 | 70.39 | 77.74 | 0.59 |  |
| -0.4 | 91 | 42 | 137 | 13 | 87.5 | 76.54 | 80.57 | 0.62 |  |
| -0.3 | 88 | 34 | 145 | 16 | 84.62 | 81.01 | 82.33 | 0.64 | ** |
| -0.2 | 85 | 27 | 152 | 19 | 81.73 | 84.92 | 83.75 | 0.66 |  |
| -0.1 | 80 | 23 | 156 | 24 | 76.92 | 87.15 | 83.39 | 0.64 |  |
| 0 | 76 | 21 | 158 | 28 | 73.08 | 88.27 | 82.69 | 0.62 |  |
| 0.1 | 72 | 16 | 163 | 32 | 69.23 | 91.06 | 83.04 | 0.63 |  |
| 0.2 | 63 | 12 | 167 | 41 | 60.58 | 93.3 | 81.27 | 0.59 |  |
| 0.3 | 61 | 10 | 169 | 43 | 58.65 | 94.41 | 81.27 | 0.59 |  |
| 0.4 | 52 | 6 | 173 | 52 | 50 | 96.65 | 79.51 | 0.56 |  |
| 0.5 | 46 | 4 | 175 | 58 | 44.23 | 97.77 | 78.09 | 0.53 |  |
| 0.6 | 42 | 2 | 177 | 62 | 40.38 | 98.88 | 77.39 | 0.52 |  |
| 0.7 | 35 | 0 | 179 | 69 | 33.65 | 100 | 75.62 | 0.49 |  |
| 0.8 | 31 | 0 | 179 | 73 | 29.81 | 100 | 74.2 | 0.46 |  |
| 0.9 | 19 | 0 | 179 | 85 | 18.27 | 100 | 69.96 | 0.35 |  |
| 1 | 11 | 0 | 179 | 93 | 10.58 | 100 | 67.14 | 0.26 |  |
| IBK | | | | | | | | | |
| 0 | 104 | 179 | 0 | 0 | 100 | 0 | 36.75 | 0 |  |
| 0.1 | 89 | 40 | 139 | 15 | 85.58 | 77.65 | 80.57 | 0.61 |  |
| 0.2 | 89 | 34 | 145 | 15 | 85.58 | 81.01 | 82.69 | 0.65 |  |
| 0.3 | 86 | 22 | 157 | 18 | 82.69 | 87.71 | 85.87 | 0.7 |  |
| 0.4 | 70 | 10 | 169 | 34 | 67.31 | 94.41 | 84.45 | 0.66 |  |
| 0.5 | 69 | 9 | 170 | 35 | 66.35 | 94.97 | 84.45 | 0.66 |  |
| 0.6 | 65 | 8 | 171 | 39 | 62.5 | 95.53 | 83.39 | 0.64 |  |
| 0.7 | 46 | 3 | 176 | 58 | 44.23 | 98.32 | 78.45 | 0.54 |  |
| 0.8 | 42 | 3 | 176 | 62 | 40.38 | 98.32 | 77.03 | 0.51 |  |
| 0.9 | 40 | 2 | 177 | 64 | 38.46 | 98.88 | 76.68 | 0.51 |  |
| 1 | 18 | 1 | 178 | 86 | 17.31 | 99.44 | 69.26 | 0.32 |  |
