# Supplementary material for: Improved Method for Linear B-Cell Epitope Prediction Using Antigen’s Primary Sequence
Source: PLoS One. 2013 May 7;8(5):e62216. doi: 10.1371/journal.pone.0062216 (PMC3646881; doi:10.1371/journal.pone.0062216)
Supplement: Table S18 — The performance of SVM/IBK models developed on Lbtope_Fixed_non_redundant dataset using amino acid composition. These models were developed using 5-fold cross-validation on 90% data and tested on remaining 10% data. (DOC) [file pone.0062216.s021.doc]

**Table S18. The performance of SVM/IBK models developed on Lbtope_Fixed_non_redundant dataset using amino acid composition. These models were developed using 5-fold cross-validation on 90% data and tested on remaining 10% data.**

| **SVM** | | | | | | | | | |
| --- | --- | --- | --- | --- | --- | --- | --- | --- | --- |
| **Thres** | **TP** | **FP** | **TN** | **FN** | **Sen** | **Spec** | **Accuracy** | **MCC** |  |
| -1 | 763 | 759 | 7 | 2 | 99.74 | 0.91 | 50.29 | 0.04 |  |
| -0.9 | 760 | 756 | 10 | 5 | 99.35 | 1.31 | 50.29 | 0.03 |  |
| -0.8 | 753 | 746 | 20 | 12 | 98.43 | 2.61 | 50.49 | 0.04 |  |
| -0.7 | 748 | 736 | 30 | 17 | 97.78 | 3.92 | 50.82 | 0.05 |  |
| -0.6 | 727 | 723 | 43 | 38 | 95.03 | 5.61 | 50.29 | 0.01 |  |
| -0.5 | 708 | 696 | 70 | 57 | 92.55 | 9.14 | 50.82 | 0.03 |  |
| -0.4 | 682 | 651 | 115 | 83 | 89.15 | 15.01 | 52.06 | 0.06 |  |
| -0.3 | 651 | 592 | 174 | 114 | 85.1 | 22.72 | 53.89 | 0.1 |  |
| -0.2 | 615 | 530 | 236 | 150 | 80.39 | 30.81 | 55.58 | 0.13 |  |
| -0.1 | 542 | 423 | 343 | 223 | 70.85 | 44.78 | 57.81 | 0.16 |  |
| 0 | 454 | 327 | 439 | 311 | 59.35 | 57.31 | 58.33 | 0.17 | ** |
| 0.1 | 371 | 230 | 536 | 394 | 48.5 | 69.97 | 59.24 | 0.19 |  |
| 0.2 | 246 | 149 | 617 | 519 | 32.16 | 80.55 | 56.37 | 0.15 |  |
| 0.3 | 176 | 84 | 682 | 589 | 23.01 | 89.03 | 56.04 | 0.16 |  |
| 0.4 | 103 | 42 | 724 | 662 | 13.46 | 94.52 | 54.02 | 0.14 |  |
| 0.5 | 64 | 23 | 743 | 701 | 8.37 | 97 | 52.71 | 0.12 |  |
| 0.6 | 30 | 10 | 756 | 735 | 3.92 | 98.69 | 51.34 | 0.08 |  |
| 0.7 | 15 | 5 | 761 | 750 | 1.96 | 99.35 | 50.69 | 0.06 |  |
| 0.8 | 5 | 1 | 765 | 760 | 0.65 | 99.87 | 50.29 | 0.04 |  |
| 0.9 | 2 | 1 | 765 | 763 | 0.26 | 99.87 | 50.1 | 0.01 |  |
| 1 | 0 | 0 | 766 | 765 | 0 | 100 | 50.03 | 0 |  |
| IBK | | | | | | | | | |
| 0 | 765 | 766 | 0 | 0 | 100 | 0 | 49.97 | 0 |  |
| 0.1 | 698 | 667 | 99 | 67 | 91.24 | 12.92 | 52.06 | 0.07 |  |
| 0.2 | 688 | 643 | 123 | 77 | 89.93 | 16.06 | 52.97 | 0.09 |  |
| 0.3 | 644 | 556 | 210 | 121 | 84.18 | 27.42 | 55.78 | 0.14 |  |
| 0.4 | 558 | 470 | 296 | 207 | 72.94 | 38.64 | 55.78 | 0.12 |  |
| 0.5 | 479 | 384 | 382 | 286 | 62.61 | 49.87 | 56.24 | 0.13 |  |
| 0.6 | 291 | 197 | 569 | 474 | 38.04 | 74.28 | 56.17 | 0.13 |  |
| 0.7 | 202 | 113 | 653 | 563 | 26.41 | 85.25 | 55.85 | 0.14 |  |
| 0.8 | 142 | 67 | 699 | 623 | 18.56 | 91.25 | 54.93 | 0.14 |  |
| 0.9 | 111 | 53 | 713 | 654 | 14.51 | 93.08 | 53.82 | 0.12 |  |
| 1 | 111 | 53 | 713 | 654 | 14.51 | 93.08 | 53.82 | 0.12 |  |
