# Supplementary material for: Improved Method for Linear B-Cell Epitope Prediction Using Antigen’s Primary Sequence
Source: PLoS One. 2013 May 7;8(5):e62216. doi: 10.1371/journal.pone.0062216 (PMC3646881; doi:10.1371/journal.pone.0062216)
Supplement: Table S19 — The performance of SVM/IBK models developed on Lbtope_Fixed_non_redundant dataset using composition-transition. These models were developed using 5-fold cross-validation on 90% data and tested on remaining 10% data. (DOC) [file pone.0062216.s022.doc]

**Table S19. The performance of SVM/IBK models developed on Lbtope_Fixed_non_redundant dataset using composition-transition. These models were developed using 5-fold cross-validation on 90% data and tested on remaining 10% data.**

| **SVM** | | | | | | | | | |
| --- | --- | --- | --- | --- | --- | --- | --- | --- | --- |
| **Thres** | **TP** | **FP** | **TN** | **FN** | **Sen** | **Spec** | **Accuracy** | **MCC** |  |
| -1 | 733 | 713 | 53 | 32 | 95.82 | 6.92 | 51.34 | 0.06 |  |
| -0.9 | 714 | 691 | 75 | 51 | 93.33 | 9.79 | 51.53 | 0.06 |  |
| -0.8 | 697 | 663 | 103 | 68 | 91.11 | 13.45 | 52.25 | 0.07 |  |
| -0.7 | 674 | 636 | 130 | 91 | 88.1 | 16.97 | 52.51 | 0.07 |  |
| -0.6 | 649 | 594 | 172 | 116 | 84.84 | 22.45 | 53.63 | 0.09 |  |
| -0.5 | 612 | 552 | 214 | 153 | 80 | 27.94 | 53.95 | 0.09 |  |
| -0.4 | 581 | 513 | 253 | 184 | 75.95 | 33.03 | 54.47 | 0.1 |  |
| -0.3 | 547 | 477 | 289 | 218 | 71.5 | 37.73 | 54.6 | 0.1 |  |
| -0.2 | 512 | 423 | 343 | 253 | 66.93 | 44.78 | 55.85 | 0.12 |  |
| -0.1 | 463 | 384 | 382 | 302 | 60.52 | 49.87 | 55.19 | 0.1 |  |
| 0 | 416 | 327 | 439 | 349 | 54.38 | 57.31 | 55.85 | 0.12 | ** |
| 0.1 | 368 | 285 | 481 | 397 | 48.1 | 62.79 | 55.45 | 0.11 |  |
| 0.2 | 315 | 243 | 523 | 450 | 41.18 | 68.28 | 54.74 | 0.1 |  |
| 0.3 | 279 | 191 | 575 | 486 | 36.47 | 75.07 | 55.78 | 0.13 |  |
| 0.4 | 230 | 165 | 601 | 535 | 30.07 | 78.46 | 54.28 | 0.1 |  |
| 0.5 | 197 | 131 | 635 | 568 | 25.75 | 82.9 | 54.34 | 0.11 |  |
| 0.6 | 162 | 104 | 662 | 603 | 21.18 | 86.42 | 53.82 | 0.1 |  |
| 0.7 | 129 | 79 | 687 | 636 | 16.86 | 89.69 | 53.3 | 0.1 |  |
| 0.8 | 92 | 58 | 708 | 673 | 12.03 | 92.43 | 52.25 | 0.07 |  |
| 0.9 | 66 | 43 | 723 | 699 | 8.63 | 94.39 | 51.53 | 0.06 |  |
| 1 | 52 | 26 | 740 | 713 | 6.8 | 96.61 | 51.73 | 0.08 |  |
| IBK | | | | | | | | | |
| 0 | 765 | 766 | 0 | 0 | 100 | 0 | 49.97 | 0 |  |
| 0.1 | 765 | 766 | 0 | 0 | 100 | 0 | 49.97 | 0 |  |
| 0.2 | 754 | 746 | 20 | 11 | 98.56 | 2.61 | 50.56 | 0.04 |  |
| 0.3 | 709 | 691 | 75 | 56 | 92.68 | 9.79 | 51.21 | 0.04 |  |
| 0.4 | 604 | 541 | 225 | 161 | 78.95 | 29.37 | 54.15 | 0.1 |  |
| 0.5 | 410 | 347 | 419 | 355 | 53.59 | 54.7 | 54.15 | 0.08 |  |
| 0.6 | 185 | 147 | 619 | 580 | 24.18 | 80.81 | 52.51 | 0.06 |  |
| 0.7 | 51 | 42 | 724 | 714 | 6.67 | 94.52 | 50.62 | 0.02 |  |
| 0.8 | 21 | 4 | 762 | 744 | 2.75 | 99.48 | 51.14 | 0.09 |  |
| 0.9 | 7 | 1 | 765 | 758 | 0.92 | 99.87 | 50.42 | 0.05 |  |
| 1 | 2 | 1 | 765 | 763 | 0.26 | 99.87 | 50.1 | 0.01 |  |
