# Supplementary material for: Improved Method for Linear B-Cell Epitope Prediction Using Antigen’s Primary Sequence
Source: PLoS One. 2013 May 7;8(5):e62216. doi: 10.1371/journal.pone.0062216 (PMC3646881; doi:10.1371/journal.pone.0062216)
Supplement: Table S20 — The performance of SVM/IBK models developed on Lbtope_Fixed_non_redundant dataset using composition-transition. These models were developed using 5-fold cross-validation on 90% data and tested on remaining 10% data. (DOC) [file pone.0062216.s023.doc]

**Table S20. The performance of SVM/IBK models developed on Lbtope_Fixed_non_redundant dataset using composition-transition. These models were developed using 5-fold cross-validation on 90% data and tested on remaining 10% data.**

| **SVM** | | | | | | | | | |
| --- | --- | --- | --- | --- | --- | --- | --- | --- | --- |
| **Thres** | **TP** | **FP** | **TN** | **FN** | **Sen** | **Spec** | **Accuracy** | **MCC** |  |
| -1 | 734 | 672 | 94 | 31 | 95.95 | 12.27 | 54.08 | 0.15 |  |
| -0.9 | 726 | 648 | 118 | 39 | 94.9 | 15.4 | 55.13 | 0.17 |  |
| -0.8 | 715 | 624 | 142 | 50 | 93.46 | 18.54 | 55.98 | 0.18 |  |
| -0.7 | 701 | 588 | 178 | 64 | 91.63 | 23.24 | 57.41 | 0.2 |  |
| -0.6 | 684 | 558 | 208 | 81 | 89.41 | 27.15 | 58.26 | 0.21 |  |
| -0.5 | 665 | 521 | 245 | 100 | 86.93 | 31.98 | 59.44 | 0.23 |  |
| -0.4 | 642 | 471 | 295 | 123 | 83.92 | 38.51 | 61.2 | 0.25 |  |
| -0.3 | 615 | 428 | 338 | 150 | 80.39 | 44.13 | 62.25 | 0.26 |  |
| -0.2 | 577 | 383 | 383 | 188 | 75.42 | 50 | 62.7 | 0.26 |  |
| -0.1 | 545 | 344 | 422 | 220 | 71.24 | 55.09 | 63.16 | 0.27 |  |
| 0 | 504 | 302 | 464 | 261 | 65.88 | 60.57 | 63.23 | 0.26 | ** |
| 0.1 | 466 | 277 | 489 | 299 | 60.92 | 63.84 | 62.38 | 0.25 |  |
| 0.2 | 413 | 238 | 528 | 352 | 53.99 | 68.93 | 61.46 | 0.23 |  |
| 0.3 | 371 | 206 | 560 | 394 | 48.5 | 73.11 | 60.81 | 0.22 |  |
| 0.4 | 317 | 171 | 595 | 448 | 41.44 | 77.68 | 59.57 | 0.21 |  |
| 0.5 | 263 | 140 | 626 | 502 | 34.38 | 81.72 | 58.07 | 0.18 |  |
| 0.6 | 213 | 114 | 652 | 552 | 27.84 | 85.12 | 56.5 | 0.16 |  |
| 0.7 | 172 | 100 | 666 | 593 | 22.48 | 86.95 | 54.74 | 0.12 |  |
| 0.8 | 122 | 73 | 693 | 643 | 15.95 | 90.47 | 53.23 | 0.1 |  |
| 0.9 | 90 | 53 | 713 | 675 | 11.76 | 93.08 | 52.45 | 0.08 |  |
| 1 | 70 | 37 | 729 | 695 | 9.15 | 95.17 | 52.19 | 0.08 |  |
| IBK | | | | | | | | | |
| 0 | 765 | 766 | 0 | 0 | 100 | 0 | 49.97 | 0 |  |
| 0.1 | 625 | 403 | 363 | 140 | 81.7 | 47.39 | 64.53 | 0.31 |  |
| 0.2 | 625 | 403 | 363 | 140 | 81.7 | 47.39 | 64.53 | 0.31 |  |
| 0.3 | 623 | 400 | 366 | 142 | 81.44 | 47.78 | 64.6 | 0.31 |  |
| 0.4 | 610 | 364 | 402 | 155 | 79.74 | 52.48 | 66.1 | 0.33 |  |
| 0.5 | 491 | 223 | 543 | 274 | 64.18 | 70.89 | 67.54 | 0.35 |  |
| 0.6 | 358 | 130 | 636 | 407 | 46.8 | 83.03 | 64.92 | 0.32 |  |
| 0.7 | 329 | 110 | 656 | 436 | 43.01 | 85.64 | 64.34 | 0.32 |  |
| 0.8 | 327 | 110 | 656 | 438 | 42.75 | 85.64 | 64.21 | 0.31 |  |
| 0.9 | 326 | 110 | 656 | 439 | 42.61 | 85.64 | 64.14 | 0.31 |  |
| 1 | 326 | 110 | 656 | 439 | 42.61 | 85.64 | 64.14 | 0.31 |  |
