# Supplementary material for: Improved Method for Linear B-Cell Epitope Prediction Using Antigen’s Primary Sequence
Source: PLoS One. 2013 May 7;8(5):e62216. doi: 10.1371/journal.pone.0062216 (PMC3646881; doi:10.1371/journal.pone.0062216)
Supplement: Table S21 — The performance of SVM/IBK models developed on Lbtope_Fixed_non_redundant dataset using dipeptide composition. These models were developed using 5-fold cross-validation on 90% data and tested on remaining 10% data. (DOC) [file pone.0062216.s024.doc]

**Table S21. The performance of SVM/IBK models developed on Lbtope_Fixed_non_redundant dataset using dipeptide composition. These models were developed using 5-fold cross-validation on 90% data and tested on remaining 10% data.**

| **SVM** | | | | | | | | | |
| --- | --- | --- | --- | --- | --- | --- | --- | --- | --- |
| **Thres** | **TP** | **FP** | **TN** | **FN** | **Sen** | **Spec** | **Accuracy** | **MCC** |  |
| -1 | 756 | 718 | 48 | 9 | 98.82 | 6.27 | 52.51 | 0.13 |  |
| -0.9 | 749 | 696 | 70 | 16 | 97.91 | 9.14 | 53.49 | 0.15 |  |
| -0.8 | 743 | 670 | 96 | 22 | 97.12 | 12.53 | 54.8 | 0.18 |  |
| -0.7 | 728 | 629 | 137 | 37 | 95.16 | 17.89 | 56.5 | 0.21 |  |
| -0.6 | 712 | 585 | 181 | 53 | 93.07 | 23.63 | 58.33 | 0.23 |  |
| -0.5 | 689 | 523 | 243 | 76 | 90.07 | 31.72 | 60.88 | 0.27 |  |
| -0.4 | 664 | 485 | 281 | 101 | 86.8 | 36.68 | 61.72 | 0.27 |  |
| -0.3 | 638 | 437 | 329 | 127 | 83.4 | 42.95 | 63.16 | 0.29 |  |
| -0.2 | 590 | 390 | 376 | 175 | 77.12 | 49.09 | 63.1 | 0.27 |  |
| -0.1 | 549 | 335 | 431 | 216 | 71.76 | 56.27 | 64.01 | 0.28 |  |
| 0 | 503 | 276 | 490 | 262 | 65.75 | 63.97 | 64.86 | 0.3 | ** |
| 0.1 | 451 | 227 | 539 | 314 | 58.95 | 70.37 | 64.66 | 0.3 |  |
| 0.2 | 398 | 184 | 582 | 367 | 52.03 | 75.98 | 64.01 | 0.29 |  |
| 0.3 | 350 | 154 | 612 | 415 | 45.75 | 79.9 | 62.83 | 0.27 |  |
| 0.4 | 287 | 130 | 636 | 478 | 37.52 | 83.03 | 60.29 | 0.23 |  |
| 0.5 | 232 | 112 | 654 | 533 | 30.33 | 85.38 | 57.87 | 0.19 |  |
| 0.6 | 184 | 84 | 682 | 581 | 24.05 | 89.03 | 56.56 | 0.17 |  |
| 0.7 | 150 | 67 | 699 | 615 | 19.61 | 91.25 | 55.45 | 0.16 |  |
| 0.8 | 113 | 47 | 719 | 652 | 14.77 | 93.86 | 54.34 | 0.14 |  |
| 0.9 | 84 | 32 | 734 | 681 | 10.98 | 95.82 | 53.43 | 0.13 |  |
| 1 | 60 | 18 | 748 | 705 | 7.84 | 97.65 | 52.78 | 0.12 |  |
| IBK | | | | | | | | | |
| 0 | 765 | 766 | 0 | 0 | 100 | 0 | 49.97 | 0 |  |
| 0.1 | 521 | 240 | 526 | 244 | 68.1 | 68.67 | 68.39 | 0.37 |  |
| 0.2 | 520 | 240 | 526 | 245 | 67.97 | 68.67 | 68.32 | 0.37 |  |
| 0.3 | 511 | 238 | 528 | 254 | 66.8 | 68.93 | 67.86 | 0.36 |  |
| 0.4 | 506 | 232 | 534 | 259 | 66.14 | 69.71 | 67.93 | 0.36 |  |
| 0.5 | 501 | 232 | 534 | 264 | 65.49 | 69.71 | 67.6 | 0.35 |  |
| 0.6 | 455 | 189 | 577 | 310 | 59.48 | 75.33 | 67.41 | 0.35 |  |
| 0.7 | 440 | 179 | 587 | 325 | 57.52 | 76.63 | 67.08 | 0.35 |  |
| 0.8 | 439 | 178 | 588 | 326 | 57.39 | 76.76 | 67.08 | 0.35 |  |
| 0.9 | 438 | 178 | 588 | 327 | 57.25 | 76.76 | 67.02 | 0.35 |  |
| 1 | 438 | 178 | 588 | 327 | 57.25 | 76.76 | 67.02 | 0.35 |  |
