# Supplementary material for: Improved Method for Linear B-Cell Epitope Prediction Using Antigen’s Primary Sequence
Source: PLoS One. 2013 May 7;8(5):e62216. doi: 10.1371/journal.pone.0062216 (PMC3646881; doi:10.1371/journal.pone.0062216)
Supplement: Table S22 — The performance of SVM/IBK model developed on Lbtope_Variable_non_redundant dataset using amino acid composition. These models were developed using 5-fold cross-validation on 90% data and tested on remaining 10%. (DOC) [file pone.0062216.s025.doc]

**Table S22.The performance of SVM/IBK model developed on Lbtope_Variable_non_redundant dataset using amino acid composition. These models were developed using 5-fold cross-validation on 90% data and tested on remaining 10%.**

| **SVM** | | | | | | | | | |
| --- | --- | --- | --- | --- | --- | --- | --- | --- | --- |
| **Thres** | **TP** | **FP** | **TN** | **FN** | **Sen** | **Spec** | **Accuracy** | **MCC** |  |
| -1 | 685 | 804 | 246 | 118 | 85.31 | 23.43 | 50.24 | 0.11 |  |
| -0.9 | 663 | 746 | 304 | 140 | 82.57 | 28.95 | 52.19 | 0.13 |  |
| -0.8 | 624 | 691 | 359 | 179 | 77.71 | 34.19 | 53.05 | 0.13 |  |
| -0.7 | 597 | 638 | 412 | 206 | 74.35 | 39.24 | 54.45 | 0.14 |  |
| -0.6 | 551 | 586 | 464 | 252 | 68.62 | 44.19 | 54.78 | 0.13 |  |
| -0.5 | 512 | 528 | 522 | 291 | 63.76 | 49.71 | 55.8 | 0.13 |  |
| -0.4 | 481 | 474 | 576 | 322 | 59.9 | 54.86 | 57.04 | 0.15 |  |
| -0.3 | 453 | 421 | 629 | 350 | 56.41 | 59.9 | 58.39 | 0.16 | ** |
| -0.2 | 411 | 369 | 681 | 392 | 51.18 | 64.86 | 58.93 | 0.16 |  |
| -0.1 | 374 | 329 | 721 | 429 | 46.58 | 68.67 | 59.09 | 0.16 |  |
| 0 | 335 | 284 | 766 | 468 | 41.72 | 72.95 | 59.42 | 0.15 |  |
| 0.1 | 307 | 243 | 807 | 496 | 38.23 | 76.86 | 60.12 | 0.16 |  |
| 0.2 | 283 | 202 | 848 | 520 | 35.24 | 80.76 | 61.04 | 0.18 |  |
| 0.3 | 247 | 167 | 883 | 556 | 30.76 | 84.1 | 60.98 | 0.18 |  |
| 0.4 | 215 | 134 | 916 | 588 | 26.77 | 87.24 | 61.04 | 0.18 |  |
| 0.5 | 178 | 106 | 944 | 625 | 22.17 | 89.9 | 60.55 | 0.17 |  |
| 0.6 | 145 | 89 | 961 | 658 | 18.06 | 91.52 | 59.69 | 0.14 |  |
| 0.7 | 118 | 68 | 982 | 685 | 14.69 | 93.52 | 59.36 | 0.14 |  |
| 0.8 | 100 | 55 | 995 | 703 | 12.45 | 94.76 | 59.09 | 0.13 |  |
| 0.9 | 80 | 43 | 1007 | 723 | 9.96 | 95.9 | 58.66 | 0.12 |  |
| 1 | 53 | 32 | 1018 | 750 | 6.6 | 96.95 | 57.8 | 0.08 |  |
| IBK | | | | | | | | | |
| 0 | 803 | 1050 | 0 | 0 | 100 | 0 | 43.34 | 0 |  |
| 0.1 | 672 | 746 | 304 | 131 | 83.69 | 28.95 | 52.67 | 0.15 |  |
| 0.2 | 658 | 700 | 350 | 145 | 81.94 | 33.33 | 54.4 | 0.17 |  |
| 0.3 | 580 | 551 | 499 | 223 | 72.23 | 47.52 | 58.23 | 0.2 |  |
| 0.4 | 394 | 332 | 718 | 409 | 49.07 | 68.38 | 60.01 | 0.18 |  |
| 0.5 | 358 | 286 | 764 | 445 | 44.58 | 72.76 | 60.55 | 0.18 |  |
| 0.6 | 321 | 242 | 808 | 482 | 39.98 | 76.95 | 60.93 | 0.18 |  |
| 0.7 | 147 | 101 | 949 | 656 | 18.31 | 90.38 | 59.15 | 0.13 |  |
| 0.8 | 111 | 61 | 989 | 692 | 13.82 | 94.19 | 59.36 | 0.14 |  |
| 0.9 | 106 | 53 | 997 | 697 | 13.2 | 94.95 | 59.53 | 0.14 |  |
| 1 | 106 | 53 | 997 | 697 | 13.2 | 94.95 | 59.53 | 0.14 |  |
