# Supplementary material for: Improved Method for Linear B-Cell Epitope Prediction Using Antigen’s Primary Sequence
Source: PLoS One. 2013 May 7;8(5):e62216. doi: 10.1371/journal.pone.0062216 (PMC3646881; doi:10.1371/journal.pone.0062216)
Supplement: Table S23 — The performance of SVM/IBK models developed on Lbtope_Variable_non_redundant dataset using composition-transition. These models were developed using 5-fold cross-validation on 90% data and tested on remaining 10% data. (DOC) [file pone.0062216.s026.doc]

**Table S23. The performance of SVM/IBK models developed on Lbtope_Variable_non_redundant dataset using composition-transition. These models were developed using 5-fold cross-validation on 90% data and tested on remaining 10% data.**

| **SVM** | | | | | | | | | |
| --- | --- | --- | --- | --- | --- | --- | --- | --- | --- |
| **Thres** | **TP** | **FP** | **TN** | **FN** | **Sen** | **Spec** | **Accuracy** | **MCC** |  |
| -1 | 691 | 758 | 292 | 112 | 86.05 | 27.81 | 53.05 | 0.17 |  |
| -0.9 | 630 | 617 | 433 | 173 | 78.46 | 41.24 | 57.37 | 0.21 |  |
| -0.8 | 568 | 509 | 541 | 235 | 70.73 | 51.52 | 59.85 | 0.22 |  |
| -0.7 | 518 | 423 | 627 | 285 | 64.51 | 59.71 | 61.79 | 0.24 |  |
| -0.6 | 469 | 348 | 702 | 334 | 58.41 | 66.86 | 63.19 | 0.25 | ** |
| -0.5 | 433 | 302 | 748 | 370 | 53.92 | 71.24 | 63.73 | 0.25 |  |
| -0.4 | 398 | 267 | 783 | 405 | 49.56 | 74.57 | 63.73 | 0.25 |  |
| -0.3 | 366 | 238 | 812 | 437 | 45.58 | 77.33 | 63.57 | 0.24 |  |
| -0.2 | 344 | 211 | 839 | 459 | 42.84 | 79.9 | 63.84 | 0.25 |  |
| -0.1 | 318 | 183 | 867 | 485 | 39.6 | 82.57 | 63.95 | 0.25 |  |
| 0 | 295 | 158 | 892 | 508 | 36.74 | 84.95 | 64.06 | 0.25 |  |
| 0.1 | 263 | 140 | 910 | 540 | 32.75 | 86.67 | 63.3 | 0.23 |  |
| 0.2 | 242 | 124 | 926 | 561 | 30.14 | 88.19 | 63.03 | 0.23 |  |
| 0.3 | 225 | 104 | 946 | 578 | 28.02 | 90.1 | 63.19 | 0.23 |  |
| 0.4 | 198 | 84 | 966 | 605 | 24.66 | 92 | 62.82 | 0.23 |  |
| 0.5 | 174 | 65 | 985 | 629 | 21.67 | 93.81 | 62.55 | 0.23 |  |
| 0.6 | 136 | 52 | 998 | 667 | 16.94 | 95.05 | 61.2 | 0.2 |  |
| 0.7 | 111 | 41 | 1009 | 692 | 13.82 | 96.1 | 60.44 | 0.18 |  |
| 0.8 | 82 | 31 | 1019 | 721 | 10.21 | 97.05 | 59.42 | 0.15 |  |
| 0.9 | 58 | 23 | 1027 | 745 | 7.22 | 97.81 | 58.55 | 0.12 |  |
| 1 | 38 | 10 | 1040 | 765 | 4.73 | 99.05 | 58.18 | 0.12 |  |
| IBK | | | | | | | | | |
| 0 | 803 | 1050 | 0 | 0 | 100 | 0 | 43.34 | 0 |  |
| 0.1 | 794 | 999 | 51 | 9 | 98.88 | 4.86 | 45.6 | 0.1 |  |
| 0.2 | 740 | 863 | 187 | 63 | 92.15 | 17.81 | 50.03 | 0.14 |  |
| 0.3 | 621 | 632 | 418 | 182 | 77.33 | 39.81 | 56.07 | 0.18 |  |
| 0.4 | 476 | 376 | 674 | 327 | 59.28 | 64.19 | 62.06 | 0.23 |  |
| 0.5 | 306 | 189 | 861 | 497 | 38.11 | 82 | 62.98 | 0.23 |  |
| 0.6 | 181 | 94 | 956 | 622 | 22.54 | 91.05 | 61.36 | 0.19 |  |
| 0.7 | 82 | 32 | 1018 | 721 | 10.21 | 96.95 | 59.36 | 0.15 |  |
| 0.8 | 46 | 13 | 1037 | 757 | 5.73 | 98.76 | 58.45 | 0.13 |  |
| 0.9 | 17 | 1 | 1049 | 786 | 2.12 | 99.9 | 57.53 | 0.1 |  |
| 1 | 5 | 0 | 1050 | 798 | 0.62 | 100 | 56.93 | 0.06 |  |
