# Supplementary material for: Improved Method for Linear B-Cell Epitope Prediction Using Antigen’s Primary Sequence
Source: PLoS One. 2013 May 7;8(5):e62216. doi: 10.1371/journal.pone.0062216 (PMC3646881; doi:10.1371/journal.pone.0062216)
Supplement: Table S24 — The performance of SVM/IBK models developed on Lbtope_Variable_non_redundant dataset using composition-transition. These models were developed using 5-fold cross-validation on 90% data and tested on remaining 10% data. (DOC) [file pone.0062216.s027.doc]

**Table S24. The performance of SVM/IBK models developed on Lbtope_Variable_non_redundant dataset using composition-transition. These models were developed using 5-fold cross-validation on 90% data and tested on remaining 10% data.**

| **SVM** | | | | | | | | | |
| --- | --- | --- | --- | --- | --- | --- | --- | --- | --- |
| **Thres** | **TP** | **FP** | **TN** | **FN** | **Sen** | **Spec** | **Accuracy** | **MCC** |  |
| -1 | 733 | 804 | 246 | 70 | 91.28 | 23.43 | 52.83 | 0.19 |  |
| -0.9 | 710 | 743 | 307 | 93 | 88.42 | 29.24 | 54.88 | 0.21 |  |
| -0.8 | 682 | 670 | 380 | 121 | 84.93 | 36.19 | 57.31 | 0.24 |  |
| -0.7 | 655 | 611 | 439 | 148 | 81.57 | 41.81 | 59.04 | 0.25 |  |
| -0.6 | 624 | 542 | 508 | 179 | 77.71 | 48.38 | 61.09 | 0.27 |  |
| -0.5 | 589 | 496 | 554 | 214 | 73.35 | 52.76 | 61.68 | 0.26 |  |
| -0.4 | 545 | 440 | 610 | 258 | 67.87 | 58.1 | 62.33 | 0.26 |  |
| -0.3 | 510 | 389 | 661 | 293 | 63.51 | 62.95 | 63.19 | 0.26 | ** |
| -0.2 | 468 | 328 | 722 | 335 | 58.28 | 68.76 | 64.22 | 0.27 |  |
| -0.1 | 429 | 278 | 772 | 374 | 53.42 | 73.52 | 64.81 | 0.27 |  |
| 0 | 388 | 224 | 826 | 415 | 48.32 | 78.67 | 65.52 | 0.28 |  |
| 0.1 | 353 | 192 | 858 | 450 | 43.96 | 81.71 | 65.35 | 0.28 |  |
| 0.2 | 306 | 156 | 894 | 497 | 38.11 | 85.14 | 64.76 | 0.27 |  |
| 0.3 | 277 | 125 | 925 | 526 | 34.5 | 88.1 | 64.87 | 0.27 |  |
| 0.4 | 233 | 97 | 953 | 570 | 29.02 | 90.76 | 64 | 0.26 |  |
| 0.5 | 206 | 83 | 967 | 597 | 25.65 | 92.1 | 63.3 | 0.24 |  |
| 0.6 | 169 | 63 | 987 | 634 | 21.05 | 94 | 62.39 | 0.23 |  |
| 0.7 | 147 | 47 | 1003 | 656 | 18.31 | 95.52 | 62.06 | 0.22 |  |
| 0.8 | 115 | 38 | 1012 | 688 | 14.32 | 96.38 | 60.82 | 0.19 |  |
| 0.9 | 86 | 29 | 1021 | 717 | 10.71 | 97.24 | 59.74 | 0.16 |  |
| 1 | 71 | 22 | 1028 | 732 | 8.84 | 97.9 | 59.31 | 0.15 |  |
| IBK | | | | | | | | | |
| 0 | 803 | 1050 | 0 | 0 | 100 | 0 | 43.34 | 0 |  |
| 0.1 | 518 | 452 | 598 | 285 | 64.51 | 56.95 | 60.23 | 0.21 |  |
| 0.2 | 517 | 440 | 610 | 286 | 64.38 | 58.1 | 60.82 | 0.22 |  |
| 0.3 | 509 | 335 | 715 | 294 | 63.39 | 68.1 | 66.06 | 0.31 |  |
| 0.4 | 267 | 120 | 930 | 536 | 33.25 | 88.57 | 64.6 | 0.27 |  |
| 0.5 | 247 | 103 | 947 | 556 | 30.76 | 90.19 | 64.44 | 0.27 |  |
| 0.6 | 238 | 90 | 960 | 565 | 29.64 | 91.43 | 64.65 | 0.27 |  |
| 0.7 | 125 | 33 | 1017 | 678 | 15.57 | 96.86 | 61.63 | 0.22 |  |
| 0.8 | 72 | 16 | 1034 | 731 | 8.97 | 98.48 | 59.69 | 0.17 |  |
| 0.9 | 64 | 13 | 1037 | 739 | 7.97 | 98.76 | 59.42 | 0.17 |  |
| 1 | 64 | 13 | 1037 | 739 | 7.97 | 98.76 | 59.42 | 0.17 |  |
