# Supplementary material for: Improved Method for Linear B-Cell Epitope Prediction Using Antigen’s Primary Sequence
Source: PLoS One. 2013 May 7;8(5):e62216. doi: 10.1371/journal.pone.0062216 (PMC3646881; doi:10.1371/journal.pone.0062216)
Supplement: Table S25 — The performance of SVM/IBK models developed on Lbtope_Variable_non_redundant dataset using dipeptide composition. These models were developed using 5-fold cross-validation on 90% data and tested on remaining 10% data. (DOC) [file pone.0062216.s028.doc]

**Table S25. The performance of SVM/IBK models developed on Lbtope_Variable_non_redundant dataset using dipeptide composition. These models were developed using 5-fold cross-validation on 90% data and tested on remaining 10% data.**

| **SVM** | | | | | | | | | |
| --- | --- | --- | --- | --- | --- | --- | --- | --- | --- |
| **Thres** | **TP** | **FP** | **TN** | **FN** | **Sen** | **Spec** | **Accuracy** | **MCC** |  |
| -1 | 789 | 956 | 94 | 14 | 98.26 | 8.95 | 47.65 | 0.15 |  |
| -0.9 | 782 | 897 | 153 | 21 | 97.38 | 14.57 | 50.46 | 0.2 |  |
| -0.8 | 771 | 847 | 203 | 32 | 96.01 | 19.33 | 52.56 | 0.23 |  |
| -0.7 | 749 | 783 | 267 | 54 | 93.28 | 25.43 | 54.83 | 0.24 |  |
| -0.6 | 722 | 710 | 340 | 81 | 89.91 | 32.38 | 57.31 | 0.26 |  |
| -0.5 | 702 | 627 | 423 | 101 | 87.42 | 40.29 | 60.71 | 0.3 |  |
| -0.4 | 669 | 549 | 501 | 134 | 83.31 | 47.71 | 63.14 | 0.32 |  |
| -0.3 | 621 | 479 | 571 | 182 | 77.33 | 54.38 | 64.33 | 0.32 |  |
| -0.2 | 579 | 408 | 642 | 224 | 72.1 | 61.14 | 65.89 | 0.33 |  |
| -0.1 | 530 | 344 | 706 | 273 | 66 | 67.24 | 66.7 | 0.33 | ** |
| 0 | 483 | 292 | 758 | 320 | 60.15 | 72.19 | 66.97 | 0.32 |  |
| 0.1 | 434 | 230 | 820 | 369 | 54.05 | 78.1 | 67.67 | 0.33 |  |
| 0.2 | 379 | 174 | 876 | 424 | 47.2 | 83.43 | 67.73 | 0.33 |  |
| 0.3 | 319 | 134 | 916 | 484 | 39.73 | 87.24 | 66.65 | 0.31 |  |
| 0.4 | 265 | 96 | 954 | 538 | 33 | 90.86 | 65.79 | 0.3 |  |
| 0.5 | 214 | 70 | 980 | 589 | 26.65 | 93.33 | 64.44 | 0.27 |  |
| 0.6 | 163 | 42 | 1008 | 640 | 20.3 | 96 | 63.19 | 0.26 |  |
| 0.7 | 125 | 33 | 1017 | 678 | 15.57 | 96.86 | 61.63 | 0.22 |  |
| 0.8 | 85 | 23 | 1027 | 718 | 10.59 | 97.81 | 60.01 | 0.18 |  |
| 0.9 | 58 | 14 | 1036 | 745 | 7.22 | 98.67 | 59.04 | 0.15 |  |
| 1 | 44 | 6 | 1044 | 759 | 5.48 | 99.43 | 58.72 | 0.15 |  |
| IBK | | | | | | | | | |
| 0 | 803 | 1050 | 0 | 0 | 100 | 0 | 43.34 | 0 |  |
| 0.1 | 639 | 379 | 671 | 164 | 79.58 | 63.9 | 70.7 | 0.43 |  |
| 0.2 | 632 | 375 | 675 | 171 | 78.7 | 64.29 | 70.53 | 0.43 |  |
| 0.3 | 614 | 351 | 699 | 189 | 76.46 | 66.57 | 70.86 | 0.43 |  |
| 0.4 | 575 | 297 | 753 | 228 | 71.61 | 71.71 | 71.67 | 0.43 |  |
| 0.5 | 481 | 215 | 835 | 322 | 59.9 | 79.52 | 71.02 | 0.4 |  |
| 0.6 | 308 | 81 | 969 | 495 | 38.36 | 92.29 | 68.92 | 0.37 |  |
| 0.7 | 265 | 60 | 990 | 538 | 33 | 94.29 | 67.73 | 0.36 |  |
| 0.8 | 257 | 57 | 993 | 546 | 32 | 94.57 | 67.46 | 0.35 |  |
| 0.9 | 254 | 57 | 993 | 549 | 31.63 | 94.57 | 67.3 | 0.35 |  |
| 1 | 254 | 57 | 993 | 549 | 31.63 | 94.57 | 67.3 | 0.35 |  |
