# Supplementary material for: Improved Method for Linear B-Cell Epitope Prediction Using Antigen’s Primary Sequence
Source: PLoS One. 2013 May 7;8(5):e62216. doi: 10.1371/journal.pone.0062216 (PMC3646881; doi:10.1371/journal.pone.0062216)
Supplement: Table S26 — The performance of Chen’s AAP model on Lbtope_Fixed data. (DOC) [file pone.0062216.s029.doc]

**Table S26. The performance of Chen’s AAP model on Lbtope_Fixed data.**

| **Thres** | **TP** | **FP** | **TN** | **FN** | **Sen** | **Spec** | **Accuracy** | **MCC** |
| --- | --- | --- | --- | --- | --- | --- | --- | --- |
| **-1** | 11123 | 18430 | 2159 | 940 | 92.21 | 10.49 | 40.68 | 0.04 |
| **-0.9** | 10781 | 17786 | 2803 | 1282 | 89.37 | 13.61 | 41.6 | 0.04 |
| **-0.8** | 10399 | 17015 | 3574 | 1664 | 86.21 | 17.36 | 42.79 | 0.05 |
| **-0.7** | 9929 | 16129 | 4460 | 2134 | 82.31 | 21.66 | 44.07 | 0.05 |
| **-0.6** | 9428 | 15169 | 5420 | 2635 | 78.16 | 26.32 | 45.47 | 0.05 |
| **-0.5** | 8853 | 14077 | 6512 | 3210 | 73.39 | 31.63 | 47.06 | 0.05 |
| **-0.4** | 8201 | 12876 | 7713 | 3862 | 67.98 | 37.46 | 48.74 | 0.05 |
| **-0.3** | 7491 | 11682 | 8907 | 4572 | 62.1 | 43.26 | 50.22 | 0.05 |
| **-0.2** | 6812 | 10488 | 10101 | 5251 | 56.47 | 49.06 | 51.8 | 0.05 |
| **-0.1** | 6131 | 9252 | 11337 | 5932 | 50.82 | 55.06 | 53.5 | 0.06 |
| **0** | 5448 | 8146 | 12443 | 6615 | 45.16 | 60.44 | 54.79 | 0.05 |
| **0.1** | 4796 | 7012 | 13577 | 7267 | 39.76 | 65.94 | 56.27 | 0.06 |
| **0.2** | 4189 | 5981 | 14608 | 7874 | 34.73 | 70.95 | 57.57 | 0.06 |
| **0.3** | 3646 | 5029 | 15560 | 8417 | 30.22 | 75.57 | 58.82 | 0.06 |
| **0.4** | 3127 | 4174 | 16415 | 8936 | 25.92 | 79.73 | 59.85 | 0.07 |
| **0.5** | 2697 | 3433 | 17156 | 9366 | 22.36 | 83.33 | 60.8 | 0.07 |
| **0.6** | 2304 | 2829 | 17760 | 9759 | 19.1 | 86.26 | 61.45 | 0.07 |
| **0.7** | 1926 | 2294 | 18295 | 10137 | 15.97 | 88.86 | 61.93 | 0.07 |
| **0.8** | 1590 | 1839 | 18750 | 10473 | 13.18 | 91.07 | 62.29 | 0.07 |
| **0.9** | 1342 | 1443 | 19146 | 10721 | 11.12 | 92.99 | 62.75 | 0.07 |
| **1** | 1054 | 1093 | 19496 | 11009 | 8.74 | 94.69 | 62.94 | 0.07 |
