# Supplementary material for: Improved Method for Linear B-Cell Epitope Prediction Using Antigen’s Primary Sequence
Source: PLoS One. 2013 May 7;8(5):e62216. doi: 10.1371/journal.pone.0062216 (PMC3646881; doi:10.1371/journal.pone.0062216)
Supplement: Table S27 — Performance of BCPred model (20 mer) on Lbtope_Fixed dataset. (DOC) [file pone.0062216.s030.doc]

**Table S27. Performance of BCPred model (20 mer) on Lbtope_Fixed dataset.**

| **Thres** | **TP** | **FP** | **TN** | **FN** | **Sen** | **Spec** | **Accuracy** | **MCC** |  |
| --- | --- | --- | --- | --- | --- | --- | --- | --- | --- |
| **0** | 12063 | 20589 | 0 | 0 | 100 | 0 | 36.94 | 0 |  |
| **0.1** | 10046 | 15793 | 4796 | 2017 | 83.28 | 23.29 | 45.46 | 0.08 |  |
| **0.2** | 9141 | 13919 | 6670 | 2922 | 75.78 | 32.4 | 48.42 | 0.09 |  |
| **0.3** | 8398 | 12519 | 8070 | 3665 | 69.62 | 39.2 | 50.43 | 0.09 |  |
| **0.4** | 7719 | 11316 | 9273 | 4344 | 63.99 | 45.04 | 52.04 | 0.09 |  |
| **0.5** | 7102 | 10198 | 10391 | 4961 | 58.87 | 50.47 | 53.57 | 0.09 | ** (Weka default) |
| **0.6** | 6442 | 9122 | 11467 | 5621 | 53.4 | 55.69 | 54.85 | 0.09 |  |
| **0.7** | 5752 | 8005 | 12584 | 6311 | 47.68 | 61.12 | 56.16 | 0.09 |  |
| **0.8** | 4992 | 6760 | 13829 | 7071 | 41.38 | 67.17 | 57.64 | 0.09 |  |
| **0.9** | 4027 | 5145 | 15444 | 8036 | 33.38 | 75.01 | 59.63 | 0.09 |  |
| **1** | 1193 | 1408 | 19181 | 10870 | 9.89 | 93.16 | 62.4 | 0.05 |  |
