# Supplementary material for: Improved Method for Linear B-Cell Epitope Prediction Using Antigen’s Primary Sequence
Source: PLoS One. 2013 May 7;8(5):e62216. doi: 10.1371/journal.pone.0062216 (PMC3646881; doi:10.1371/journal.pone.0062216)
Supplement: Table S28 — The performance of SVM models developed on Lbtope_Fixed dataset tested on Chen dataset. (DOC) [file pone.0062216.s031.doc]

**Table S28. The performance of SVM models developed on Lbtope_Fixed dataset tested on Chen dataset.**

| **Thres** | **TP** | **FP** | **TN** | **FN** | **Sen** | **Spec** | **Accuracy** | **MCC** |
| --- | --- | --- | --- | --- | --- | --- | --- | --- |
| **-1** | 818 | 849 | 23 | 54 | 93.81 | 2.64 | 48.22 | -0.09 |
| **-0.9** | 779 | 835 | 37 | 93 | 89.33 | 4.24 | 46.79 | -0.12 |
| **-0.8** | 766 | 811 | 61 | 106 | 87.84 | 7.00 | 47.42 | -0.09 |
| **-0.7** | 743 | 781 | 91 | 129 | 85.21 | 10.44 | 47.82 | -0.07 |
| **-0.6** | 707 | 748 | 124 | 165 | 81.08 | 14.22 | 47.65 | -0.06 |
| **-0.5** | 681 | 693 | 179 | 191 | 78.10 | 20.53 | 49.31 | -0.02 |
| **-0.4** | 649 | 634 | 238 | 223 | 74.43 | 27.29 | 50.86 | 0.02 |
| **-0.3** | 617 | 559 | 313 | 255 | 70.76 | 35.89 | 53.33 | 0.07 |
| **-0.2** | 576 | 474 | 398 | 296 | 66.06 | 45.64 | 55.85 | 0.12 |
| **-0.1** | 535 | 391 | 481 | 337 | 61.35 | 55.16 | 58.26 | 0.17 |
| **0** | 495 | 318 | 554 | 377 | 56.77 | 63.53 | 60.15 | 0.20 |
| **0.1** | 446 | 262 | 610 | 426 | 51.15 | 69.95 | 60.55 | 0.21 |
| **0.2** | 405 | 199 | 673 | 467 | 46.44 | 77.18 | 61.81 | 0.25 |
| **0.3** | 366 | 157 | 715 | 506 | 41.97 | 82.00 | 61.98 | 0.26 |
| **0.4** | 331 | 113 | 759 | 541 | 37.96 | 87.04 | 62.50 | 0.29 |
| **0.5** | 297 | 85 | 787 | 575 | 34.06 | 90.25 | 62.16 | 0.29 |
| **0.6** | 262 | 56 | 816 | 610 | 30.05 | 93.58 | 61.81 | 0.31 |
| **0.7** | 225 | 39 | 833 | 647 | 25.80 | 95.53 | 60.67 | 0.30 |
| **0.8** | 196 | 21 | 851 | 676 | 22.48 | 97.59 | 60.03 | 0.30 |
| **0.9** | 171 | 15 | 857 | 701 | 19.61 | 98.28 | 58.94 | 0.29 |
| **1** | 102 | 9 | 863 | 770 | 11.70 | 98.97 | 55.33 | 0.22 |
