# Supplementary material for: Improved Method for Linear B-Cell Epitope Prediction Using Antigen’s Primary Sequence
Source: PLoS One. 2013 May 7;8(5):e62216. doi: 10.1371/journal.pone.0062216 (PMC3646881; doi:10.1371/journal.pone.0062216)
Supplement: Table S29 — The performance of SVM models developed on Lbtope_Fixed dataset tested on BCPred dataset. (DOC) [file pone.0062216.s032.doc]

**Table S29. The performance of SVM models developed on Lbtope_Fixed dataset tested on BCPred dataset.**

| **Thres** | **TP** | **FP** | **TN** | **FN** | **Sen** | **Spec** | **Accuracy** | **MCC** |
| --- | --- | --- | --- | --- | --- | --- | --- | --- |
| **-1** | 655 | 679 | 22 | 46 | 93.44 | 3.14 | 48.29 | -0.08 |
| **-0.9** | 622 | 665 | 36 | 79 | 88.73 | 5.14 | 46.93 | -0.11 |
| **-0.8** | 608 | 651 | 50 | 93 | 86.73 | 7.13 | 46.93 | -0.10 |
| **-0.7** | 586 | 628 | 73 | 115 | 83.59 | 10.41 | 47.00 | -0.09 |
| **-0.6** | 556 | 605 | 96 | 145 | 79.32 | 13.69 | 46.50 | -0.09 |
| **-0.5** | 531 | 562 | 139 | 170 | 75.75 | 19.83 | 47.79 | -0.05 |
| **-0.4** | 510 | 514 | 187 | 191 | 72.75 | 26.68 | 49.71 | -0.01 |
| **-0.3** | 486 | 464 | 237 | 215 | 69.33 | 33.81 | 51.57 | 0.03 |
| **-0.2** | 449 | 408 | 293 | 252 | 64.05 | 41.80 | 52.92 | 0.06 |
| **-0.1** | 419 | 347 | 354 | 282 | 59.77 | 50.50 | 55.14 | 0.10 |
| **0** | 387 | 278 | 423 | 314 | 55.21 | 60.34 | 57.77 | 0.16 |
| **0.1** | 348 | 225 | 476 | 353 | 49.64 | 67.90 | 58.77 | 0.18 |
| **0.2** | 315 | 172 | 529 | 386 | 44.94 | 75.46 | 60.20 | 0.21 |
| **0.3** | 283 | 127 | 574 | 418 | 40.37 | 81.88 | 61.13 | 0.24 |
| **0.4** | 256 | 89 | 612 | 445 | 36.52 | 87.30 | 61.91 | 0.28 |
| **0.5** | 233 | 63 | 638 | 468 | 33.24 | 91.01 | 62.13 | 0.30 |
| **0.6** | 205 | 45 | 656 | 496 | 29.24 | 93.58 | 61.41 | 0.30 |
| **0.7** | 187 | 34 | 667 | 514 | 26.68 | 95.15 | 60.91 | 0.30 |
| **0.8** | 161 | 19 | 682 | 540 | 22.97 | 97.29 | 60.13 | 0.30 |
| **0.9** | 140 | 10 | 691 | 561 | 19.97 | 98.57 | 59.27 | 0.30 |
| **1** | 83 | 6 | 695 | 618 | 11.84 | 99.14 | 55.49 | 0.23 |
