# Supplementary material for: Improved Method for Linear B-Cell Epitope Prediction Using Antigen’s Primary Sequence
Source: PLoS One. 2013 May 7;8(5):e62216. doi: 10.1371/journal.pone.0062216 (PMC3646881; doi:10.1371/journal.pone.0062216)
Supplement: Table S30 — Performance of ABCPred model (20 mer) on Lbtope_Fixed dataset. (DOC) [file pone.0062216.s033.doc]

**Table S30. Performance of ABCPred model (20 mer) on Lbtope_Fixed** dataset.

| **Thres** | **TP** | **FP** | **TN** | **FN** | **Sen** | **Spec** | **Accuracy** | **MCC** |  |
| --- | --- | --- | --- | --- | --- | --- | --- | --- | --- |
| 0 | 12063 | 20589 | 0 | 0 | 100 | 0 | 36.94 | 0 |  |
| 0.1 | 12026 | 20516 | 73 | 37 | 99.69 | 0.35 | 37.05 | 0 |  |
| 0.2 | 11581 | 19523 | 1066 | 482 | 96 | 5.18 | 38.73 | 0.03 |  |
| 0.3 | 10214 | 16955 | 3634 | 1849 | 84.67 | 17.65 | 42.41 | 0.03 |  |
| 0.4 | 8656 | 14047 | 6542 | 3407 | 71.76 | 31.77 | 46.55 | 0.04 |  |
| 0.5 | 6580 | 10389 | 10200 | 5483 | 54.55 | 49.54 | 51.39 | 0.04 | ** (Weka default) |
| 0.6 | 4383 | 6765 | 13824 | 7680 | 36.33 | 67.14 | 55.76 | 0.04 |  |
| 0.7 | 2357 | 3476 | 17113 | 9706 | 19.54 | 83.12 | 59.63 | 0.03 |  |
| 0.8 | 819 | 1139 | 19450 | 11244 | 6.79 | 94.47 | 62.08 | 0.03 |  |
| 0.9 | 84 | 115 | 20474 | 11979 | 0.7 | 99.44 | 62.96 | 0.01 |  |
| 1 | 0 | 0 | 20589 | 12063 | 0 | 100 | 63.06 | 0 |  |
