# Supplementary material for: Improved Method for Linear B-Cell Epitope Prediction Using Antigen’s Primary Sequence
Source: PLoS One. 2013 May 7;8(5):e62216. doi: 10.1371/journal.pone.0062216 (PMC3646881; doi:10.1371/journal.pone.0062216)
Supplement: Table S31 — The performance of SVM models developed on Lbtope_Fixed and tested on ABCPred dataset. (DOC) [file pone.0062216.s034.doc]

**Table S31. The performance of SVM models developed on Lbtope_Fixed** and tested on ABCPred dataset.

| **Thres** | **TP** | **FP** | **TN** | **FN** | **Sen** | **Spec** | **Accuracy** | **MCC** |
| --- | --- | --- | --- | --- | --- | --- | --- | --- |
| -1 | 1369 | 688 | 12 | 31 | 97.79 | 1.71 | 65.76 | -0.02 |
| -0.9 | 1346 | 679 | 21 | 54 | 96.14 | 3 | 65.1 | -0.02 |
| -0.8 | 1317 | 664 | 36 | 83 | 94.07 | 5.14 | 64.43 | -0.02 |
| -0.7 | 1284 | 649 | 51 | 116 | 91.71 | 7.29 | 63.57 | -0.02 |
| -0.6 | 1234 | 617 | 83 | 166 | 88.14 | 11.86 | 62.71 | 0 |
| -0.5 | 1162 | 574 | 126 | 238 | 83 | 18 | 61.33 | 0.01 |
| -0.4 | 1078 | 531 | 169 | 322 | 77 | 24.14 | 59.38 | 0.01 |
| -0.3 | 980 | 464 | 236 | 420 | 70 | 33.71 | 57.9 | 0.04 |
| -0.2 | 879 | 391 | 309 | 521 | 62.79 | 44.14 | 56.57 | 0.07 |
| -0.1 | 782 | 329 | 371 | 618 | 55.86 | 53 | 54.9 | 0.08 |
| 0 | 690 | 268 | 432 | 710 | 49.29 | 61.71 | 53.43 | 0.1 |
| 0.1 | 580 | 201 | 499 | 820 | 41.43 | 71.29 | 51.38 | 0.12 |
| 0.2 | 490 | 147 | 553 | 910 | 35 | 79 | 49.67 | 0.14 |
| 0.3 | 410 | 106 | 594 | 990 | 29.29 | 84.86 | 47.81 | 0.15 |
| 0.4 | 343 | 71 | 629 | 1057 | 24.5 | 89.86 | 46.29 | 0.17 |
| 0.5 | 277 | 48 | 652 | 1123 | 19.79 | 93.14 | 44.24 | 0.17 |
| 0.6 | 218 | 29 | 671 | 1182 | 15.57 | 95.86 | 42.33 | 0.17 |
| 0.7 | 160 | 18 | 682 | 1240 | 11.43 | 97.43 | 40.1 | 0.15 |
| 0.8 | 117 | 12 | 688 | 1283 | 8.36 | 98.29 | 38.33 | 0.13 |
| 0.9 | 79 | 8 | 692 | 1321 | 5.64 | 98.86 | 36.71 | 0.11 |
| 1 | 40 | 3 | 697 | 1360 | 2.86 | 99.57 | 35.1 | 0.08 |
