# Supplementary material for: Improved Method for Linear B-Cell Epitope Prediction Using Antigen’s Primary Sequence
Source: PLoS One. 2013 May 7;8(5):e62216. doi: 10.1371/journal.pone.0062216 (PMC3646881; doi:10.1371/journal.pone.0062216)
Supplement: Table S32 — The performance of SVM models developed on Lbtope_Variable and tested on bcpred variable data. (DOC) [file pone.0062216.s035.doc]

**Table S32. The performance of SVM models developed on Lbtope_Variable and tested on bcpred variable data**

| **SVM** | | | | | | | | |
| --- | --- | --- | --- | --- | --- | --- | --- | --- |
| **Thres** | **TP** | **FP** | **TN** | **FN** | **Sen** | **Spec** | **Accuracy** | **MCC** |
| -1 | 1208 | 1197 | 26 | 15 | 98.77 | 2.13 | 50.45 | 0.04 |
| -0.9 | 1193 | 1174 | 49 | 30 | 97.55 | 4.01 | 50.78 | 0.04 |
| -0.8 | 1168 | 1144 | 79 | 55 | 95.5 | 6.46 | 50.98 | 0.04 |
| -0.7 | 1141 | 1095 | 128 | 82 | 93.3 | 10.47 | 51.88 | 0.07 |
| -0.6 | 1108 | 1042 | 181 | 115 | 90.6 | 14.8 | 52.7 | 0.08 |
| -0.5 | 1067 | 972 | 251 | 156 | 87.24 | 20.52 | 53.88 | 0.1 |
| -0.4 | 1019 | 900 | 323 | 204 | 83.32 | 26.41 | 54.87 | 0.12 |
| -0.3 | 969 | 819 | 404 | 254 | 79.23 | 33.03 | 56.13 | 0.14 |
| -0.2 | 911 | 726 | 497 | 312 | 74.49 | 40.64 | 57.56 | 0.16 |
| -0.1 | 843 | 632 | 591 | 380 | 68.93 | 48.32 | 58.63 | 0.18 |
| 0 | 777 | 535 | 688 | 446 | 63.53 | 56.26 | 59.89 | 0.2 |
| 0.1 | 721 | 444 | 779 | 502 | 58.95 | 63.7 | 61.32 | 0.23 |
| 0.2 | 643 | 357 | 866 | 580 | 52.58 | 70.81 | 61.69 | 0.24 |
| 0.3 | 567 | 278 | 945 | 656 | 46.36 | 77.27 | 61.82 | 0.25 |
| 0.4 | 499 | 203 | 1020 | 724 | 40.8 | 83.4 | 62.1 | 0.27 |
| 0.5 | 436 | 139 | 1084 | 787 | 35.65 | 88.63 | 62.14 | 0.29 |
| 0.6 | 374 | 95 | 1128 | 849 | 30.58 | 92.23 | 61.41 | 0.29 |
| 0.7 | 307 | 69 | 1154 | 916 | 25.1 | 94.36 | 59.73 | 0.27 |
| 0.8 | 254 | 40 | 1183 | 969 | 20.77 | 96.73 | 58.75 | 0.27 |
| 0.9 | 190 | 30 | 1193 | 1033 | 15.54 | 97.55 | 56.54 | 0.23 |
| 1 | 109 | 19 | 1204 | 1114 | 8.91 | 98.45 | 53.68 | 0.17 |
| IBK | | | | | | | | |
| 0 | 1223 | 1223 | 0 | 0 | 100 | 0 | 50 | 0 |
| 0.1 | 945 | 823 | 400 | 278 | 77.27 | 32.71 | 54.99 | 0.11 |
| 0.2 | 931 | 801 | 422 | 292 | 76.12 | 34.51 | 55.31 | 0.12 |
| 0.3 | 904 | 748 | 475 | 319 | 73.92 | 38.84 | 56.38 | 0.14 |
| 0.4 | 857 | 676 | 547 | 366 | 70.07 | 44.73 | 57.4 | 0.15 |
| 0.5 | 767 | 540 | 683 | 456 | 62.71 | 55.85 | 59.28 | 0.19 |
| 0.6 | 581 | 294 | 929 | 642 | 47.51 | 75.96 | 61.73 | 0.24 |
| 0.7 | 529 | 236 | 987 | 694 | 43.25 | 80.7 | 61.98 | 0.26 |
| 0.8 | 480 | 226 | 997 | 743 | 39.25 | 81.52 | 60.38 | 0.23 |
| 0.9 | 446 | 223 | 1000 | 777 | 36.47 | 81.77 | 59.12 | 0.2 |
| 1 | 431 | 223 | 1000 | 792 | 35.24 | 81.77 | 58.5 | 0.19 |
