# Supplementary material for: Improved Method for Linear B-Cell Epitope Prediction Using Antigen’s Primary Sequence
Source: PLoS One. 2013 May 7;8(5):e62216. doi: 10.1371/journal.pone.0062216 (PMC3646881; doi:10.1371/journal.pone.0062216)
Supplement: Table S33 — SVM results of five-fold cross validation on Lbtope_Confirm (epitope tested by at least two studies) dataset using dipeptide composition. (DOC) [file pone.0062216.s036.doc]

**Table S33. SVM results of five-fold cross validation on Lbtope_Confirm (epitope tested by at least two studies) dataset using dipeptide composition.**

| **SVM** | | | | | | | | |
| --- | --- | --- | --- | --- | --- | --- | --- | --- |
| **Thres** | **TP** | **FP** | **TN** | **FN** | **Sen** | **Spec** | **Accuracy** | **MCC** |
| -1 | 930 | 1250 | 366 | 8 | 99.15 | 22.65 | 50.74 | 0.3 |
| -0.9 | 921 | 1026 | 590 | 17 | 98.19 | 36.51 | 59.16 | 0.39 |
| -0.8 | 910 | 814 | 802 | 28 | 97.01 | 49.63 | 67.03 | 0.48 |
| -0.7 | 890 | 655 | 961 | 48 | 94.88 | 59.47 | 72.47 | 0.54 |
| -0.6 | 873 | 525 | 1091 | 65 | 93.07 | 67.51 | 76.9 | 0.59 |
| -0.5 | 854 | 436 | 1180 | 84 | 91.04 | 73.02 | 79.64 | 0.62 |
| -0.4 | 833 | 364 | 1252 | 105 | 88.81 | 77.48 | 81.64 | 0.64 |
| -0.3 | 796 | 301 | 1315 | 142 | 84.86 | 81.37 | 82.65 | 0.65 |
| -0.2 | 758 | 249 | 1367 | 180 | 80.81 | 84.59 | 83.2 | 0.65 |
| -0.1 | 708 | 204 | 1412 | 230 | 75.48 | 87.38 | 83.01 | 0.63 |
| 0 | 668 | 171 | 1445 | 270 | 71.22 | 89.42 | 82.73 | 0.62 |
| 0.1 | 614 | 131 | 1485 | 324 | 65.46 | 91.89 | 82.18 | 0.61 |
| 0.2 | 559 | 111 | 1505 | 379 | 59.59 | 93.13 | 80.81 | 0.58 |
| 0.3 | 503 | 82 | 1534 | 435 | 53.62 | 94.93 | 79.76 | 0.56 |
| 0.4 | 445 | 64 | 1552 | 493 | 47.44 | 96.04 | 78.19 | 0.52 |
| 0.5 | 391 | 49 | 1567 | 547 | 41.68 | 96.97 | 76.66 | 0.49 |
| 0.6 | 340 | 35 | 1581 | 598 | 36.25 | 97.83 | 75.22 | 0.46 |
| 0.7 | 294 | 26 | 1590 | 644 | 31.34 | 98.39 | 73.77 | 0.43 |
| 0.8 | 238 | 18 | 1598 | 700 | 25.37 | 98.89 | 71.89 | 0.39 |
| 0.9 | 168 | 12 | 1604 | 770 | 17.91 | 99.26 | 69.38 | 0.32 |
| 1 | 117 | 7 | 1609 | 821 | 12.47 | 99.57 | 67.58 | 0.27 |
