# Supplementary material for: Improved Method for Linear B-Cell Epitope Prediction Using Antigen’s Primary Sequence
Source: PLoS One. 2013 May 7;8(5):e62216. doi: 10.1371/journal.pone.0062216 (PMC3646881; doi:10.1371/journal.pone.0062216)
Supplement: Table S34 — SVM results of five-fold cross validation on Lbtope_positive_fbcpred_negative (B-cell epitope (positive) from Lbtope_Confirm dataset and random peptides (negative) from fbcpred) using dipeptide composition. (DOC) [file pone.0062216.s037.doc]

**Table S34. SVM results of five-fold cross validation on Lbtope_positive_fbcpred_negative (B-cell epitope (positive) from Lbtope_Confirm dataset and random peptides (negative) from fbcpred) using dipeptide composition.**

| **SVM** | | | | | | | | |
| --- | --- | --- | --- | --- | --- | --- | --- | --- |
| **Thres** | **TP** | **FP** | **TN** | **FN** | **Sen** | **Spec** | **Accuracy** | **MCC** |
| -1 | 936 | 823 | 111 | 2 | 99.79 | 11.88 | 55.93 | 0.25 |
| -0.9 | 935 | 777 | 157 | 3 | 99.68 | 16.81 | 58.33 | 0.29 |
| -0.8 | 935 | 740 | 194 | 3 | 99.68 | 20.77 | 60.31 | 0.33 |
| -0.7 | 933 | 670 | 264 | 5 | 99.47 | 28.27 | 63.94 | 0.4 |
| -0.6 | 927 | 590 | 344 | 11 | 98.83 | 36.83 | 67.9 | 0.45 |
| -0.5 | 918 | 507 | 427 | 20 | 97.87 | 45.72 | 71.85 | 0.51 |
| -0.4 | 910 | 425 | 509 | 28 | 97.01 | 54.5 | 75.8 | 0.57 |
| -0.3 | 898 | 339 | 595 | 40 | 95.74 | 63.7 | 79.75 | 0.63 |
| -0.2 | 878 | 253 | 681 | 60 | 93.6 | 72.91 | 83.28 | 0.68 |
| -0.1 | 839 | 185 | 749 | 99 | 89.45 | 80.19 | 84.83 | 0.7 |
| 0 | 805 | 134 | 800 | 133 | 85.82 | 85.65 | 85.74 | 0.71 |
| 0.1 | 750 | 93 | 841 | 188 | 79.96 | 90.04 | 84.99 | 0.7 |
| 0.2 | 689 | 55 | 879 | 249 | 73.45 | 94.11 | 83.76 | 0.69 |
| 0.3 | 632 | 36 | 898 | 306 | 67.38 | 96.15 | 81.73 | 0.66 |
| 0.4 | 568 | 21 | 913 | 370 | 60.55 | 97.75 | 79.11 | 0.63 |
| 0.5 | 494 | 15 | 919 | 444 | 52.67 | 98.39 | 75.48 | 0.57 |
| 0.6 | 433 | 8 | 926 | 505 | 46.16 | 99.14 | 72.6 | 0.53 |
| 0.7 | 356 | 5 | 929 | 582 | 37.95 | 99.46 | 68.64 | 0.47 |
| 0.8 | 281 | 3 | 931 | 657 | 29.96 | 99.68 | 64.74 | 0.41 |
| 0.9 | 222 | 2 | 932 | 716 | 23.67 | 99.79 | 61.65 | 0.36 |
| 1 | 135 | 1 | 933 | 803 | 14.39 | 99.89 | 57.05 | 0.28 |
