# Supplementary material for: Improved Method for Linear B-Cell Epitope Prediction Using Antigen’s Primary Sequence
Source: PLoS One. 2013 May 7;8(5):e62216. doi: 10.1371/journal.pone.0062216 (PMC3646881; doi:10.1371/journal.pone.0062216)
Supplement: Text S1 — Different B-cell epitope prediction techniques already used in earlier methods. (DOC) [file pone.0062216.s038.doc]

**Physico-chemical profile**

In the past researchers have been using several amino acid properties or indices in the prediction of linear B cell epitopes (Blythe and Flower, 2005). In this study, we have tested few properties, which are found to be effective in B cell epitope prediction (additional file 1, Table S1). These are R connectivity (Rk), Relative clustering coefficient (Rc), Relative closeness (Ro), and Relative betweenness (Rb) used and tested by Huang in 2007 (Huang et al., 2007). This makes the vector size of 20x4 for each pattern.

**Amino acid pair (AAP) propensity scale**

Chen et al observed that some amino acid pairs (AAP) or dipeptides occur more frequently in B cell epitopes than non-epitopes (Chen et al., 2007). They proposed a methodology to consider the occurrence of dipeptides in both epitope and non-epitope samples and derived a propensity scale which was used in the prediction of linear B cell epitope. By adopting the same strategy, we employed main IEDB dataset to derive the AAP propensity scale (Chen et al., 2007). Similar to dipeptide composition it also creates a vector of 400 for each pattern.

**Composition-Transition-Distribution (CTD) profile**

CTD profile has been used for the MHC-binder prediction and for the handling of variable length B cell epitopes (EL-Manzalawy et al., 2008a). It maps each amino acid into groups based on corresponding physico-chemical properties, and calculation is done on these groups instead of considering each amino acid individually. CTD contains three parts; first is the overall composition of each group, second frequency of occurrence of one group after another, i.e. transition and third is distribution or composition of each group in five fragments of the peptide. We generated vector of 108 lengths for each pattern.
